# Prevalence of Non-Celiac Gluten Sensitivity in Patients with Refractory Functional Dyspepsia: a Randomized Double-blind Placebo Controlled Trial

**DOI:** 10.1038/s41598-020-59532-z

**Published:** 2020-02-12

**Authors:** Bijan Shahbazkhani, Mohammad M. Fanaeian, Mohammad J. Farahvash, Najmeh Aletaha, Foroogh Alborzi, Luca Elli, Amirhossein Shahbazkhani, Jayran Zebardast, Mohammad Rostami-Nejad

**Affiliations:** 1 Division of Gastroenterology and Liver Diseases, Imam Khomeini Hospital Complex, Tehran University of Medical Sciences, Tehran, Iran; 2 Center for Prevention and Diagnosis of Celiac Disease, Fondazione IRCCS Ca’ Granda Ospedale Maggiore Policlinico, Milan, Italy; 3 Cognitive Science Special Linguistics, Institute of Cognitive Sciences, Tehran, Iran; 4 Gastroenterology and Liver Diseases Research Center, Research Institute for Gastroenterology and Liver Diseases, Shahid Beheshti University of Medical Sciences, Tehran, Iran

**Keywords:** Functional dyspepsia, Duodenum, Stomach

## Abstract

Refractory functional dyspepsia (RFD) is characterized by symptoms persistence in spite of medical treatment or *H. pylori* eradication. No study has yet investigated the presence of gluten-dependent RFD as a clinical presentation of Non-Celiac Gluten Sensitivity (NCGS). Patients with RFD, in whom celiac disease, wheat allergy and *H. pylori* infection had been ruled out, followed a six weeks long gluten-free diet (GFD). Symptoms were evaluated by means of visual analogue scales; patients with ≥30% improvement in at least one of the reported symptoms after GFD underwent a double-blind placebo controlled gluten challenge. Subjects were randomly divided in two groups and symptoms were evaluated after the gluten/placebo challenge. GFD responders were further followed on for 3 months to evaluate the relationship between symptoms and gluten consumption. Out of 77 patients with RFD, 50 (65%) did not respond to GFD; 27 (35%) cases showed gastrointestinal symptoms improvement while on GFD; after blind gluten ingestion, symptoms recurred in 5 cases (6.4% of patients with RFD, 18% of GFD responders) suggesting the presence of NCGS. Furthermore, such extra-intestinal symptoms as fatigue and weakness (P = 0.000), musculo-skeletal pain (P = 0.000) and headache (P = 0.002) improved in NCGS patients on GFD. Because of the high prevalence of NCGS among patients with RFD, a diagnostic/therapeutic roadmap evaluating the effect of GFD in patients with RFD seems a reasonable (and simple) approach.

## Introduction

Dyspepsia is a common symptom and a diagnostic “dilemma” in daily clinical practice^1^ affecting roughly 25% of the general population. Circa 25% of patients with dyspepsia have an underlying organic or structural disease^2–6^. According to the Rome IV criteria, functional dyspepsia (FD) is defined as the presence of post-prandial fullness and/or early satiation and/or epigastric pain/burning, during the last three months with symptoms onset at least six months before diagnosis without any evidence of organic/structural disease. The prevalence of FD is about 10.6% of the general population^7,8^.

A detailed clinical history that considers heart burn, regurgitation or biliary pains suggestive for GERD, cholecystitis or NSAIDs use^9,10^, physical examinations and also upper endoscopy are all necessary to determine the underlying cause of dyspepsia^11–15^. Patients with a negative stool test for *Helicobacter pylori* (*H. pylori*) infection, as well as those with persistent symptoms after *H. pylori* eradication, are usually administrated with proton pump inhibitors (PPIs) for 8 weeks. Tricyclic antidepressants or prokinetics can be useful in patients unresponsive to PPI therapy. In case of unresponsiveness, upper endoscopy with biopsies should be performed, and psychological therapy should be considered as well^16–20^.

Refractory FD (RFD) is defined as FD presenting symptoms continuing for at least 6 months, unresponsive to at least two medical treatments such as PPIs, prokinetics, or *H. pylori* eradication^21^.

Non-celiac gluten sensitivity (NCGS) is a syndrome characterized by gastrointestinal and extra-intestinal symptoms related to the ingestion of gluten-containing products, in patients without wheat allergy (WA) or celiac disease (CD)^22,23^. According to recent studies, the prevalence of NCGS in Western populations is 0.6–10.6%^24–27^, and females are more frequently affected, with a female/male ratio ranging from 3:1^28,29^ to 5.4:1^30^. The onset of symptoms is usually rapid after gluten ingestion (from several hours up to a few days). In patients with suspected NCGS, CD and WA are usually excluded by means of serological tests (anti-tissue transglutaminase antibodies, anti-wheat IgE respectively)^22,23^ and duodenal histology^31^. Irritable bowel syndrome (IBS) is also excluded following the Rome IV clinical criteria^32,33^. Although no NCGS bio-markers have been proposed yet, high titers of anti- gliadin antibodies (AGA-IgG) are present in up to 56.4% of patients with NCGS, thus suggesting their potential use in clinical practice^34^.

The hypothesis that FD can be caused by underlying NCGS is not fully elucidated. Furthermore, according to the Salerno criteria, the diagnosis of NCGS should be considered in patients with persistent intestinal (*e.g*. dyspepsia) and/or extra-intestinal symptoms triggered by the ingestion of gluten-containing food^35^.

Because of the lack of data investigating the presence of NCGS in patients with RFD, we performed a double-blind placebo-controlled gluten-challenge trial in Iranian patients with RFD with the aim to evaluate any presence of gluten-related dyspepsia.

## Methods

### Study population

**Figure 1:**
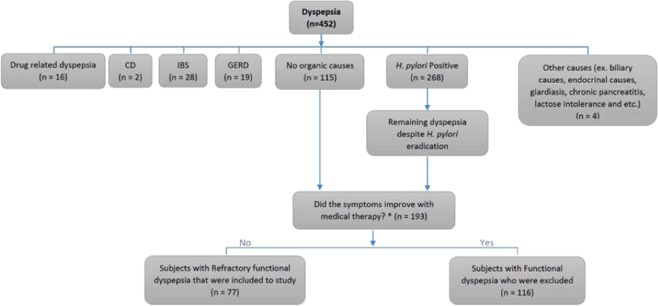
Flowchart of the described inclusion and exclusion criteria of the trial. 452 patients aged from 18 to 55 years (36.5 ± 7.3 years) with dyspepsia were evaluated for organic causes. 259 patients had an underlying cause for their symptoms and excluded, but 115 patients had no identifying causes for their symptoms. On the other hand, 78 patients with positive *H. pylori* test reported persistent symptoms despite *H. pylori* eradication; this two groups were defined against functional dyspepsia (n = 193). 193 patients with functional dyspepsia were treated with proton-pump inhibitors (PPIs) for at least 8 weeks ± Tricyclic anti-depressants (TCAs) and prokinetics for 12 weeks. 116 patients experienced symptoms relief with medical treatment and were excluded; but despite *H. pylori* eradication and medical therapy, 77 patients had persistent symptoms that defined as refractory functional dyspepsia (RFD) and were included to the study. *The medical therapy was prescribed as PPIs ± TCAs ± prokinetics. CD = Celiac disease; IBS = Irritable bowel syndrome; GERD = Gastroesophageal reflux disease.

Between February 2017 and April 2018, evaluation was carried out on patients referred for dyspepsia to the outpatient clinic of the Medical Departments, Division of Gastroenterology (Imam Khomeini Hospital Complex and Dr. Shariati Hospital, Tehran (Iran) and to the private gastroenterology clinic. FD was diagnosed accordingly to the Rome IV criteria^8^. The presence of diseases leading to dyspeptic symptoms (*i.e* celiac disease, wheat allergy, irritable bowel syndrome, lactose intolerance, diabetes mellitus, hypercalcemia, thyroid disease, biliary causes and *H. pylori* infection) were excluded by means of physical examination, serologic tests, breath tests, upper and/or lower endoscopy, abdominal ultrasound and stool tests. Patients with unremarkable tests, unresponsive to medical therapy including *H. pylori* eradication, eight weeks on PPIs, four weeks on amitriptyline and domperidone prescription, were defined as RFD (Fig. 1). AGA IgG serological titers were evaluated according to the ELISA method.

### Study protocol

**Figure 2:**
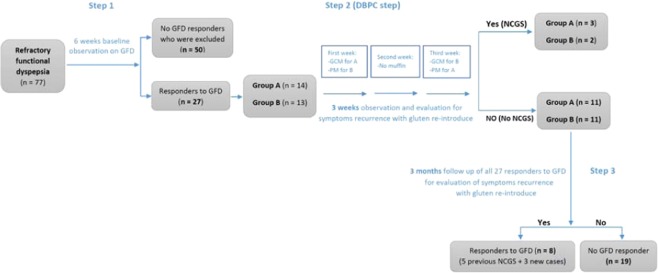
Flowchart of the trial. 77 patients with refractory functional dyspepsia entered a 6-week gluten-free diet (GFD) period and they were evaluated weekly by VAS score. The peer questionnaires were filled. 50 patients (65%) did not show any appropriate response to GFD and did not enter into the challenge phase. 27 patients (35%) showed symptoms improvement (responders) and thus they underwent a DBPC challenge. GFD was maintained for at least three more weeks. In Step 2, the GFD responders, were divided into two separate groups and blindly challenged with gluten. Finally, patients were followed for further three months in Step 3. DBPC = Double blind placebo controlled; GFD = Gluten-free diet; GCM = Gluten-containing muffin; PM = Placebo muffin (*i.e* gluten-free muffin as placebo); NCGS = Non-celiac gluten sensitivity. The evaluation is performed weekly during Steps 1, 3 and daily during Step 2.

Patients with RFD followed a 6 weeks long GFD. All pharmacologic agents were discontinued and the trial design was carried out according to the Salerno Experts criteria^35^ (Fig. 2).

Each patient received nutritional counseling about GFD from a dietician. Symptoms were weekly evaluated using a standard 10 cm long visual analogue scale (VAS). According to the Salerno Experts criteria, the patients who did not recover from their symptoms after 6 weeks on GFD were excluded from the gluten challenge. Responsiveness was defined as a 30% decrease in one to three VAS reporting the main gastrointestinal and/or extra-intestinal symptoms or at least one with no worsening of others, for at least 50% of the observation times. GFD-responders joined the second study phase (DBPC gluten challenge phase)^35^. In this study, the patient-recruiting investigator contacts a center by telephone or secure computer after a patient’s enrollment.

The Step 2 consisted in a DBPC gluten challenge with crossover. The GFD was maintained and GFD responders were randomly divided into two separate groups (group A and B based on block randomization). Their gastrointestinal (GI) and extra-intestinal symptoms were quantitatively assessed using VAS. The process involved recruiting participants in short blocks and ensuring that half of the participants within each block were allocated to treatment “A” and the other half to “B”. The baseline characteristics were similar between the two groups (Table 1). During the first week, group A received gluten-containing muffins made with coconut oil, wheat flour [including 8 gr/day gluten]^35,36^, baking soda, kosher salt, ground cinnamon, egg, banana, pure vanilla extract. Group B received gluten-free (GF) muffins as placebo, containing coconut oil, rice flour, baking soda, kosher salt, ground cinnamon, egg, banana, pure vanilla extract. In the second week, neither gluten-containing nor GF muffins were administered and patients kept their GFD (wash-out week). During the third week, group A received placebo and group B received gluten-containing muffins (crossover). As the shape and envelop of muffins were indistinguishable, and each identifiable by a unique anonymous code, both patients and investigators were blind. Finally, after the DBPC phase, all the patients were followed for 3 more months to evaluate symptoms recurrence during gluten re-introduction. Patients reporting symptoms recurrence during the ingestion of gluten-containing muffins, were diagnosed as NCGS. The symptomatic response was defined as a variation of at least 30% from the baseline value for each parameter separately. The questionnaire was also designed by a researcher and the components of the questionnaire were designed based on the study by Salerno *et al*.^35^. Then, the questionnaire was evaluated by experts and Cronbach’s alpha was calculated to be approximately 0.8.

**Table 1:** The baseline characteristics of the study groups. In the first week, group A received gluten-containing muffins and group B received gluten-free (GF) muffins as placebos; in the third week, group A received placebos and group B received gluten-containing muffins.

Variables		Overall	Group A	Group B
Number		n = 27	n = 14	n = 13
Gender		Male = 11 Female = 16	Male = 6 Female = 8	Male = 5 Female = 8
Age range		18‒55 yrs	18‒55 yrs	18‒55 yrs
Ethnic		white	white	white
Symptoms recurrence with gluten re-introduce	Yes	n = 5 (18.5%)	n = 3 (21.5%)	n = 2 (15.4%)
	No	n = 22 (81.5%)	n = 11 (78.5%)	n = 11 (84.6%)

### Statistical analysis

The results and VAS mean scores were analyzed by χ2 and Mann Whiteny’s U tests, respectively using SPSS rel. 16. Progressions of symptoms and carry-over effects for both groups were examined by Repeated Measures Using Mixed test. In this study a p value less than 0.05 was recognized significant. A repeated measurements mixed design in analysis was applied for carry-over effects.

### Informed consent statement

All the study participants provided their written informed consent prior to their study enrolment.

### CONSORT 2010 statement

The CONSORT 2010 Statement has been adopted.

### Ethical approval and informed consent

The study was performed in accordance with appropriate guidelines and reviewed and approved by the Local Ethics Committee of the Imam Khomeini Hospital (approval no.: **IR.TUMS.IKHC.REC.1395.1675**) and every enrolled patient gave his/her consent to the study participation (Supplementary File 3). Also, the clinical trial was registered as “The effect of gluten-free diet on gastrointestinal symptoms of individuals with idiopathic dyspepsia who referred to the gastroenterology clinics of Imam Khomeini Hospital complex and Dr. Shariati Hospital in 2017–2018” under the following number**: IRCT2017031833112N1 (22-04-2017)**. The methods were carried out in accordance with the relevant guidelines and regulations; and also, for nocebo effect reduction a “contextualized informed consent” was also obtained.

## Results

452 patients aged between 18 and 55 years (36.5 ± 7.3 years) with dyspepsia were evaluated: 259 were excluded because of the presence of an underlying disorder related to the clinical picture (16 patients with drug-related dyspepsia, 2 with celiac disease, 28 with IBS, 19 with GERD, 268 with *H. pylori* infection and 4 with other diseases). 115 patients were left with no apparent causes and 78 patients with positive *H. pylori* test reported symptoms permanence despite *H. pylori* eradication; these subjects were defined as affected by FD (n = 193).

193 FD patients were treated with proton pump inhibitors (PPIs) for at least 8 weeks ± Tricyclic anti-depressants (TCAs) and prokinetics for 12 weeks. 116 patients presented symptoms relief after medical treatment and were excluded; however, 77 patients had persistent symptoms despite *H. pylori* eradication and medical therapy and were defined as RFD (Fig. 1).

**Figure 3:**
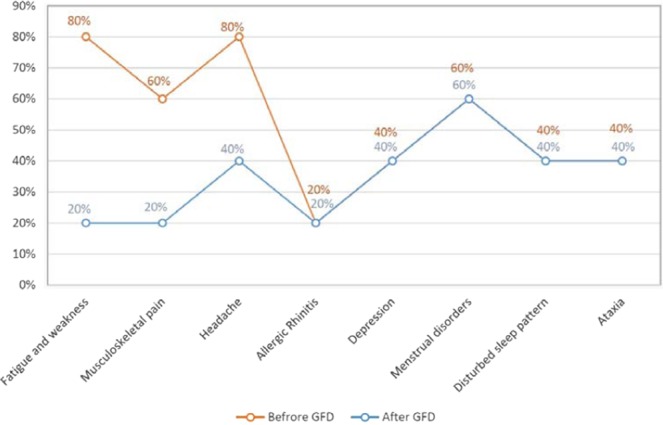
The frequency of extra-intestinal symptoms before and after GFD in 5 patients with NCGS. There were no changes in allergic rhinitis, depression, menstrual disorder, disturbed sleep patterns and ataxia. On the other hand, the patients reported impressive improvement in some symptoms such as fatigue and weakness (P. value = 0.000, P. value = 0.002) and musculo-skeletal pain (P. value = 0.000, P. value = 0.002) in more than half the time of the DBPC trial period. GFD = Gluten-free diet.

**Figure 4:**
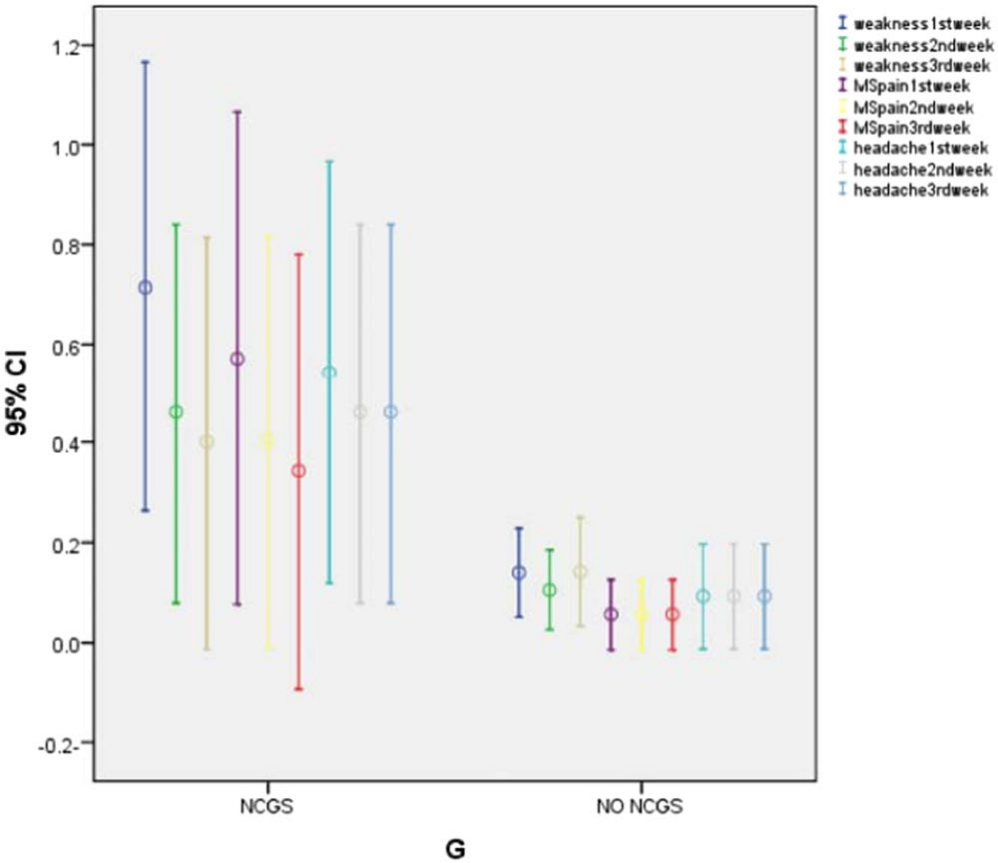
Comparison of extra-intestinal symptoms between NCGS vs. non-NCGS patients at different phases according to the questionnaire score. NCGS = Non-celiac gluten sensitivity; MS = Musculo-skeletal.

Seventy-seven patients with RFD underwent the 6 weeks long GFD. Fifty (65%) patients did not report any clinical improvement while on GFD and were excluded from the blind gluten challenge; 27 (35%) patients showed a symptomatic improvement (GFD responders) and they joined the DBPC phase, according to the Salerno criteria. At the end of Step 2 (*i.e* DBPC gluten challenge), 5 patients reported intestinal (*i.e* dyspepsia) and/or extra-intestinal symptoms’ recurrence after blind gluten ingestion (18.5% of the GFD responders and 6.4% of the patients with RFD) (Fig. 2). The sequence of gluten/placebo administration did not influence the result of the gluten challenge. NCGS was more frequent in women than men (4:1) and the mean age of the patients was 29 years (18–40 years). The most common GI symptoms were post-prandial fullness (in all patients) and epigastric pain/burning (80%). Furthermore, fatigue/headache (80%), musculo-skeletal pain and menstrual disorders (60%) were the most common extra-intestinal symptoms in NCGS patients. The serum AGA-IgG titer was high in one patient (20%) with NCGS. The overall characteristics of NCGS *vs*. non-NCGS patients are presented in Table 2. Moreover, the comparison of common extra-GI symptoms for patients before and after GFD is presented in Table 3, Figs. 3 and 4; these data show that several extra-GI symptoms, such as fatigue and weakness (P = 0.000), musculo-skeletal pain (P = 0.000) and headache (P = 0.002) improved in NCGS patients on GFD.

**Table 2:** The overall characteristics comparison of NCGS vs. non-NCGS GFD responders. Epigastric pain or burning plus post-prandial fullness have been reported as the most common GI symptoms among NCGS patients; on the other hand, fatigue, weakness and musculo-skeletal pain were the most common extra- intestinal symptoms. AGA (IgG) = Anti gliadin antibody (IgG); GI = gastro-intestinal; NCGS = Non-celiac gluten sensitivity; GFD = Gluten-free diet.

Variables		Overall (n = 27)	NCGS (n = 5, 18.5%)	No NCGS (n = 22, 81.5%)
Mean age (s.d.)		36.5 (18-55 y/o)	29 (18–40 y/o)	36.5 (18–55 y/o)
Gender	Male	11 (40.7%)	1 (20%)	10 (45.5%)
	Female	16 (59.2%)	4 (80%)	12 (54.5%)
GI symptoms	Epigastric pain/burning + post-prandial fullness + early satiation	1 (3.7%)	1 (20%)	0
	Epigastric pain/burning + post-prandial fullness	14 (51.8%)	3 (60%)	11 (50%)
	Epigastric pain/burning + early satiation	0	0	0
	Post-prandial fullness + early satiation	1 (3.7%)	0	1 (4.5%)
	Epigastric pain/burning	9 (33.3%)	0	9 (41%)
	Post-prandial fullness	1 (3.7%)	1 (20%)	0
	Early satiation	1 (3.7%)	0	1 (4.5%)
Concurrent autoimmune disease		0	0	0
Family history of celiac disease		1 (3.7%)	1 (20%)	0
Fatigue and weakness		9 (33.3%)	4 (80%)	5 (22.7%)
Musculo-skeletal pain		6 (22.2%)	3 (60%)	3 (13.6%)
Headache		6 (22.2%)	4 (80%)	2 (9%)
Asthma		0	0	0
Allergic rhinitis		2 (7.4%)	1 (20%)	1 (4.5%)
Depression		4 (14.8%)	2 (40%)	2 (9%)
Menstrual disorders		3 (11.1%)	3 (60%)	0
Disturbed sleep pattern		2 (7.4%)	2 (40%)	0
Ataxia		2 (7.4%)	2 (40%)	0
High serum AGA(IgG) level		4 (14.8%)	1 (20%)	3 (13.6%)

**Table 3:** Comparison of extra-intestinal symptoms of NCGS vs. non-NCGS patients at different phases according to the questionnaire score.

Group Statistics				95% Confidence Interval of the Difference		Sig. (2-tailed)
Time	G	Mean	Std Deviation	Lower	Upper	
Fatigue and Weakness 1^st^ week	NCGS	/714	/184	/32422	/82920	/000
	NO NCGS	/1376	/043			
MS pain 1^st^ week	NCGS	/571	/202	/2827	/7510	/000
	NO NCGS	/055	/034			
Headache 1^st^ week	NCGS	/543	/173	/1792	/7247	/002
	NO NCGS	/091	/050			
Fatigue and Weakness 2^nd^ week	NCGS	/460	/155	/13557	/57837	/002
	NO NCGS	/103	/039			
MS pain 2^nd^ week	NCGS	/403	/169	/1356	/5610	/002
	NO NCGS	/055	/034			
Headache 2^nd^ week	NCGS	/460	/155	/1047	/6335	/007
	NO NCGS	/091	/050			
Fatigue and Weakness 3^rd^ week	NCGS	/400	/169	−/0194	/5406	/067
	NO NCGS	/139	/0533			
MS pain 3^rd^ week	NCGS	/343	/178	/0697	/5069	/011
	NO NCGS	/055	/034			
Headache 3^rd^ week	NCGS	/460	/155	/1047	/6335	/007
	NO NCGS	/091	/050			

NCGS = Non-celiac gluten sensitivity; GI = Gastrointestinal symptoms; MS = Musculo-skeletal.

All 27 GFD responders were followed up for three months after the DBPC challenge and questionnaires were filled in on a weekly basis. In addition to 5 patients with NCGS, other 3 reported symptoms improvement on GFD and recurrence after ingesting gluten-containing food, despite they had shown “negative” at the DBPC challenge.

## Discussion

Our findings show that, among patients with RFD, 6.5% present a symptomatic relapse during a blind gluten challenge and therefore could be categorized as with NCGS. Moreover, during the follow-up period, some patients responding to GFD without a positive result at the DBPC gluten challenge, reported symptomatic relief after gluten ingestion increasing the prevalence of patients with suspected “gluten-related RFD”.

Recently, general practitioners and professionals have to deal with a large number of patients following some GFD, often without any medical reasons or indications. As a result, the diagnosis of NCGS becomes essential for the appropriate management of such patients and avoidance of unnecessary and expensive diets^23,37–39^. In addition, if a large number of patients with unexplained GI symptoms or IBS could be correctly treated with dietary modifications, this would reduce the need for medication with resulting direct and indirect economic benefits^40^.

According to previous studies, gluten is a relevant factor in the development of GI symptoms in patients with FD, such as in RFD^23,37,38^. Elli *et al*. in their multi-center DBPC gluten challenge study evaluated the prevalence of NCGS in patients with functional GI symptoms. In their study, out of 134 patients, including 77 (57%) IBS and 22 (16.4%) dyspeptic patients, completing a three weeks long period on GFD, 98 patients reported significant symptoms improvement: 53 (54.1%) IBS and 17 (17.3%) dyspeptic patients. They underwent a gluten challenge; finally, NCGS was confirmed in 28 subjects: 18 (64.3%) with IBS and 4 (14.3%) with dyspepsia^38^. Compared with the study by Elli *et al*., our results show higher prevalence of NCGS (18.5%) among the GFD responders.

Conversely, Dale *et al*. have shown that gluten challenges and controlled food challenges with a DBPC design are unable to detect most of the patients with suspected NCGS^41^; these findings, as well as those from other studies, suggest that, in addition to gluten, other factors (such as, fructans and amylase trypsin inhibitors) are involved in a complex mechanism at the basis of GI functional symptoms^36^.

Looking at the clinical features of NCGS patients, our results show that the presence of fullness and epigastric pain/burning, as GI symptoms, and fatigue/headache, as extra-GI symptoms, are more frequent in NCGS patients than in other study groups. Conversely, Carroccio *et al*. have shown that anemia, weight loss and a history of food allergy were more frequently reported in patients with a gluten-related disorder^39^. This dissimilarity among studies may be due to both self-imposed dietary restrictions and unbalanced diets started by patients and the heterogeneity of the enrolled patients. Comparable to our results, Biesiekierski *et al*.^23^ have reported a recurrence of symptoms in the group undergoing a re-challenge with gluten as compared to those receiving placebo.

Based on the studies recently available, about 56% of NCGS cases present high serum AGA-IgG titers^34^. Indeed, these markers may provide a diagnostic clue for NCGS among patients with RFD. In our study, the AGA (IgG) titer was high in 20% of NCGS patients. Although, the AGA (IgG) titer was not reported as a specific serologic marker for the diagnosis of NCGS, it may be a diagnostic tool for NCGS diagnosis in patients with RFD.

Recent studies have indicated the role of eosinophils in the pathogenesis of functional dyspepsia. Eosinophil infiltration in the duodenal mucosa with >22/5 high power fields (HPF) indicates an inflammatory response leading to persistence of gastrointestinal symptoms^42–46^. Eosinophils may cause sensory nerves stimulation and visceral symptoms through granules release, cytokines secretion such as interleukin IL-5, IL-4, IL-13 and mast cell activation in the duodenal mucosa of patients with FD^43,47,48^.

Furthermore, eosinophil infiltration in the duodenal and rectal mucosa of NCGS patients has been recently described^49^.

As our study focused on the symptomatic/clinical response of the patients, we could not unfortunately define any histological characteristics.

According to our findings, RFD may be linked to gluten ingestion. Although intervention with fermentable oligo-/di-/mono-saccharides and polyols (FODMAPs) could not be ruled out in our trial, we did not find any identifiable reasons for the extra-intestinal symptoms decrease in GFD responders^36,50^. On the other hand, the patients had no limitations for use of FODMAPs-containing products during all the study steps and they had to be on GFD. Thus, symptoms improvement could result from gluten exposure. Moreover, we followed the patients up for three months after the gluten challenge. During this phase, in addition to the 5 NCGS patients, we found 3 patients who reported symptoms recurrence after the ingestion of gluten-containing products and symptoms relief with GFD. The presence of these “late” responders possibly suggests that the Salerno criteria miss some NCGS patients.

Since NCGS is uncommon in the general population and a small sample size may influence the results of our study, multi-center studies with larger sample sizes are needed to clarify this point.

## Conclusions

Because of the high prevalence of NCGS among patients with RFD (6.5%), a gluten challenge or, at least, the evaluation of symptomatic responsiveness to GFD in RFD patients seems a reasonable (and simple) approach.

## Supplementary information

Supplementary Dataset 1.

Supplementary Dataset 2.

Supplementary Dataset 3.
