# Supplementary material for: Prevalence of Non-Celiac Gluten Sensitivity in Patients with Refractory Functional Dyspepsia: a Randomized Double-blind Placebo Controlled Trial
Source: Sci Rep. 2020 Feb 12;10:2401. doi: 10.1038/s41598-020-59532-z (PMC7016109; doi:10.1038/s41598-020-59532-z)
Supplement: Supplementary file 1 — Supplementary Dataset 1. [file 41598_2020_59532_MOESM1_ESM.pdf]

Prevalence of Non-Celiac Gluten Sensitivity in patients with Refractory Functional dyspepsia; a randomized double-blind placebo controlled trial

Bijan Shahbazkhani, Mohammad M. Fanaeian*, Mohammad J. Farahvash, Najmeh Aletaha, Foroogh Alborzi, Luca Elli, Amirhossein Shahbazkhani, Jayran Zebardast, Mohammad Rostami-Nejad

Bijan Shahbazkhani, Mohammad M. Fanaeian, Mohammad J. Farahvash, Najmeh Aletaha, Foroogh Alborzi, Amirhossein Shahbazkhani; Division of Gastroenterology and Liver Diseases, Imam Komeini Hospoital Complex, Tehran University of Medical Sciences, Tehran, Iran

Luca Elli; Center for Prevention and Diagnosis of Celiac Disease, Fondazione IRCCS Ca' Granda Ospedale Maggiore Policlinico, Milan, Italy

Jayran Zebardast; Cognitive Science Special Linguistics, Institute of Cognitive Sciences, Tehran, Iran

Mohammad Rostami-Nejad; Gastroenterology and Liver Diseases Research Center, Research Institute for Gastroenterology and Liver Diseases, Shahid Beheshti University of Medical Sciences, Tehran, Iran

Trial number: SREP-19-07193A

***Correspondence to: Mohammad M. Fanaeian, MD**.
Division of Gastroenterology and Liver Diseases,
Imam Komeini Hospoital Complex,
Tehran University of Medical Sciences, Tehran, Iran
E-Mail: Mohammadfanaeian@gmail.com; Tel: +989123530970

Step 1

6 weeks Gluten free diet

questionnaires for

77 subjects

STEP 1- 6 weeks GFD

Patients name **code:**

Age:

Gender: ***male*** ***female***

Address:

GI Symptoms : ***1. Epigastric pain/burning*** ***2. Post prandial*** ***3. Early satiety***

Serologic evaluation: ***tTGA (IgA):*** ***Total IgA:*** ***AGA (IgG):*** ***Anti wheat (IgE):***

EGD report : ***Marsh :***

Step 1- Six weeks follow up under GFD

Month weeks	***GI symptoms***
W1	
W2	
W3	
W4	
W5	
W6	

Step 2

3 weeks double blind placebo control

trial questionnaires for

27 subjects

Patients name: A

code: 1

Age: 37

Gender: *male* *female*

Address: Iran- Noshahr

Accompany autoimmune diseases: (DM, Hashimoto thyroiditis, Vitiligo, Adison, Herpetiform dermatitis) *Yes ....* *No*

Family history of Celiac disease: *Yes* *No*

Family history of same symptoms in first degree: *Yes* *No*

GI Symptoms : *1. Epigastric pain/burning* *2. Post prandial fullness* *3. Early satiety*

Extra GI symptoms :

1.Fatigue & weakness *2.musculoskeletal pain* *3.headache* *4.allergic rhinitis* *5.deppression* *6.menstrual disorder*

7.disturbed sleep pattern *8.ataxia*

Serologic evaluation: *tTGA (IgA):* *3.4* *Total IgA:* *190* *AGA (IgG):* *8* *Anti wheat specific (IgE):* *< 0.35 KUI/L*

Symptoms improvement

GI Symptoms : 1 2 3

Extra GI symptoms : 1 2 3 4 5 6 7 8

EGD report: *Normal* *Marsh 0*

Step2- Symptoms recurrence with gluten reintroduction

First week

Week days	GI symptoms	Extra GI symptoms
D1	1,2	1,2,3,6
D2	1,2	1,2,3,6
D3	1,2	1,2,3,6
D4	1,2	1,2,3,6
D5	1,2	1,2,3,6
D6	1,2	1,2,3,6
D7	1,2	1,2,3,6

AGA (IgG): 4.9

Stool calprotectin: 72

Second week

Week days	GI symptoms	Extra GI symptoms
D1	1,2	1,2,3,6
D2	1,2	1,2,3,6
D3	No	1,2,3,6
D4	No	6
D5	No	6
D6	No	6
D7	No	6

Third week

Week days	GI symptoms	Extra GI symptoms
D1	1,2	3,6
D2	1,2	3,6
D3	No	6
D4	No	6
D5	No	6
D6	No	6
D7	No	6

AGA (IgG): 12

Stool calprotectin: 50

Step3- Symptoms recurrence with GCP & symptoms improvement with GFD

First month

Month weeks	GI symptoms	Extra GI symptoms
W1 GCP	++	+++
W2 GFD	-	+
W3 GFD	-	+
W4 GFD	-	+

Second month

Month weeks	GI symptoms	Extra GI symptoms
W1 GCP	++	+++
W2 GFD	-	+
W3 GFD	-	+
W4 GFD	-	+

Third month

Month weeks	GI symptoms	Extra GI symptoms
W1 GCP	++	+++
W2 GFD	-	+
W3 GFD	-	+
W4 GFD	-	+

Patients name: A

code: 2

Age: 34

Gender: male female

Address: Iran- Tehran

Accompany autoimmune diseases: (DM, Hashimoto thyroiditis, Vitiligo, Adison, Herpetiform dermatitis) Yes .... No

Family history of Celiac disease: Yes No

Family history of same symptoms in first degree: Yes No

GI Symptoms : 1. Epigastric pain/burning 2. Post prandial fullness 3. Early satiety

Extra GI symptoms :

1.Fatigue & weakness 2.musculoskeletal pain 3.headache 4.allergic rhinitis 5.deppression 6.menstrual disorder

7.disturbed sleep pattern 8.ataxia

Serologic evaluation: *tTGA (IgA):* 2 *Total IgA:* 170 *AGA (IgG):* 3 *Anti wheat specific (IgE):* < 0.35 KUI/L

Symptoms improvement

GI Symptoms : 1 2 3

Extra GI symptoms : 1 2 3 4 5 6 7 8

EGD report: Mild antral erythema *Marsh* 0

Step2- Symptoms recurrence with gluten reintroduction

First week

Week days	GI symptoms	Extra GI symptoms
D1	1,2	1,2,3,4,5,6,7,8
D2	1,2	1,2,3,4,5,6,7,8
D3	1,2	1,2,3,4,5,6,7,8
D4	1,2	1,2,3,4,5,6,7,8
D5	1,2	1,2,3,4,5,6,7,8
D6	1,2	1,2,3,4,5,6,7,8
D7	1,2	1,2,3,4,5,6,7,8

AGA (IgG): 3

Stool calprotectin: 8

Second week

Week days	GI symptoms	Extra GI symptoms
D1	No	3,4,5,6,7,8
D2	No	3,4,5,6,7,8
D3	No	3,4,5,6,7,8
D4	No	3,4,5,6,7,8
D5	No	3,4,5,6,7,8
D6	No	3,4,5,6,7,8
D7	No	3,4,5,6,7,8

Third week

Week days	GI symptoms	Extra GI symptoms
D1	No	3,4,5,6,7,8
D2	No	3,4,5,6,7,8
D3	No	3,4,5,6,7,8
D4	No	3,4,5,6,7,8
D5	No	3,4,5,6,7,8
D6	No	3,4,5,6,7,8
D7	No	3,4,5,6,7,8

*AGA (IgG):*3.5

Stool calprotectin: 10

Step3- Symptoms recurrence with GCP & symptoms improvement with GFD

First month

Month weeks	GI symptoms	Extra GI symptoms
W1 GCP	++	++++++++
W2 GFD	-	++++++
W3 GFD	-	++++++
W4 GFD	-	++++++

Second month

Month weeks	GI symptoms	Extra GI symptoms
W1 GCP	++	++++++++
W2 GFD	-	++++++
W3 GFD	-	++++++
W4 GFD	-	++++++

Third month

Month weeks	GI symptoms	Extra GI symptoms
W1 GCP	++	++++++++
W2 GFD	-	++++++
W3 GFD	-	++++++
W4 GFD	-	++++++

Patients name: A

code: 3

Age: 37

Gender: *male* *female*

Address: Iran- Tehran

Accompany autoimmune diseases: (DM, Hashimoto thyroiditis, Vitiligo, Adison, Herpetiform dermatitis) **Yes ....** ***No***

Family history of Celiac disease: *Yes* **No**

Family history of same symptoms in first degree: *Yes* **No**

GI Symptoms : ***1. Epigastric pain/burning*** ***2. Post prandial fullness*** ***3. Early satiety***

Extra GI symptoms :

1.Fatigue & weakness ***2.musculoskeletal pain*** ***3.headache*** ***4.allergic rhinitis*** ***5.deppression*** ***6.menstrual disorder***

7.disturbed sleep pattern ***8.ataxia***

Serologic evaluation: ***tTGA (IgA):*** *3.5* ***Total IgA:*** *317* ***AGA (IgG):*** *3* ***Anti wheat specific (IgE):*** *< 0.35 KUI/L*

Symptoms improvement

GI Symptoms : 1 2 3

Extra GI symptoms : 1 2 3 4 5 6 7 8

EGD report: *Normal* ***Marsh*** *0*

Step2- Symptoms recurrence with gluten reintroduction

First week

Week days	GI symptoms	Extra GI symptoms
D1	1	1,3
D2	1	1,3
D3	1	1,3
D4	1	1,3
D5	1	1,3
D6	1	1,3
D7	1	1,3

AGA (IgG): *15*

Stool calprotectin: *12*

Second week

Week days	GI symptoms	Extra GI symptoms
D1	No	3
D2	No	3
D3	No	3
D4	No	3
D5	No	3
D6	No	3
D7	No	3

Third week

Week days	GI symptoms	Extra GI symptoms
D1	No	3
D2	No	3
D3	No	3
D4	No	3
D5	No	3
D6	No	3
D7	No	3

AGA (IgG):14.5

Stool calprotectin: *8.5*

Step3- Symptoms recurrence with GCP & symptoms improvement with GFD

First month

Month weeks	GI symptoms	Extra GI symptoms
W1 GCP	++	++
W2 GFD	-	+
W3 GFD	-	+
W4 GFD	-	+

Second month

Month weeks	GI symptoms	Extra GI symptoms
W1 GCP	++	++
W2 GFD	-	+
W3 GFD	-	+
W4 GFD	-	+

Third month

Month weeks	GI symptoms	Extra GI symptoms
W1 GCP	++	++
W2 GFD	-	+
W3 GFD	-	+
W4 GFD	-	+

Patients name: B

code: 4

Age: 32

Gender: *male* *female*

Address: Iran- Tehran

Accompany autoimmune diseases: (DM, Hashimoto thyroiditis, Vitiligo, Adison, Herpetiform dermatitis) *Yes ....* *No*

Family history of Celiac disease: *Yes* *No*

Family history of same symptoms in first degree: *Yes* *No*

GI Symptoms : *1. Epigastric pain/burning* *2. Post prandial fullness* *3. Early satiety*

Extra GI symptoms :

1.Fatigue & weakness *2.musculoskeletal pain* *3.headache* *4.allergic rhinitis* *5.deppression* *6.menstrual disorder*

7.disturbed sleep pattern *8.ataxia*

Serologic evaluation: *tTGA (IgA): 1* *Total IgA: 80* *AGA (IgG): 4* *Anti wheat specific (IgE): < 0.35 KUI/L*

Symptoms improvement

GI Symptoms : 1 2 3

Extra GI symptoms : 1 2 3 4 5 6 7 8

EGD report: Normal *Marsh 0*

Step2- Symptoms recurrence with gluten reintroduction

First week

Week days	GI symptoms	Extra GI symptoms
D1	No	No
D2	No	No
D3	No	No
D4	No	No
D5	No	No
D6	No	No
D7	No	No

AGA (IgG): 3.9

Stool calprotectin: 0.01

Second week

Week days	GI symptoms	Extra GI symptoms
D1	No	No
D2	No	No
D3	No	No
D4	No	No
D5	No	No
D6	No	No
D7	No	No

Third week

Week days	GI symptoms	Extra GI symptoms
D1	2	No
D2	2	No
D3	2	No
D4	2	No
D5	2	No
D6	2	No
D7	2	No

AGA (IgG):4.8

Stool calprotectin: 22

Step3- Symptoms recurrence with GCP & symptoms improvement with GFD

First month

Month weeks	GI symptoms	Extra GI symptoms
W1 GCP	+	-
W2 GFD	-	-
W3 GFD	-	-
W4 GFD	-	-

Second month

Month weeks	GI symptoms	Extra GI symptoms
W1 GCP	+	-
W2 GFD	-	-
W3 GFD	-	-
W4 GFD	-	-

Third month

Month weeks	GI symptoms	Extra GI symptoms
W1 GCP	+	-
W2 GFD	-	-
W3 GFD	-	-
W4 GFD	-	-

Patients name: B

code: 5

Age: 24

Gender: *male* *female*

Address: Iran- Tehran

Accompany autoimmune diseases: (DM, Hashimoto thyroiditis, Vitiligo, Adison, Herpetiform dermatitis) *Yes ....* *No*

Family history of Celiac disease: *Yes* *No*

Family history of same symptoms in first degree: *Yes* *No*

GI Symptoms : *1. Epigastric pain/burning* *2. Post prandial fullness* *3. Early satiety*

Extra GI symptoms :

1.Fatigue & weakness *2.musculoskeletal pain* *3.headache* *4.allergic rhinitis* *5.deppression* *6.menstrual disorder* *7.disturbed sleep pattern* *8.ataxia*

Serologic evaluation: *tTGA (IgA):* *1* *Total IgA:* *207* *AGA (IgG):* *2.3* *Anti wheat specific (IgE):* *< 0.35 KUI/L*

Symptoms improvement

GI Symptoms : 1 2 3

Extra GI symptoms : 1 2 3 4 5 6 7 8

EGD report: *Patchy mild antral erythema* *Marsh 0*

Step2- Symptoms recurrence with gluten reintroduction

First week

Week days	GI symptoms	Extra GI symptoms
D1	1,2,3	1,2,3,5,6,7,8
D2	1,2,3	1,2,3,5,6,7,8
D3	1,2,3	1,2,3,5,6,7,8
D4	1,2,3	1,2,3,5,6,7,8
D5	1,2,3	1,2,3,5,6,7,8
D6	1,2,3	1,2,3,5,6,7,8
D7	1,2,3	1,2,3,5,6,7,8

AGA (IgG): 6.2

Stool calprotectin: 96

Second week

Week days	GI symptoms	Extra GI symptoms
D1	2,3	1,2,5,6,7,8
D2	2,3	1,2,5,6,7,8
D3	2,3	1,2,5,6,7,8
D4	2,3	1,2,5,6,7,8
D5	2,3	1,2,5,6,7,8
D6	2,3	1,2,5,6,7,8
D7	2,3	1,2,5,6,7,8

Third week

Week days	GI symptoms	Extra GI symptoms
D1	2,3	1,2,5,6,7,8
D2	2,3	1,2,5,6,7,8
D3	2,3	1,2,5,6,7,8
D4	2,3	1,2,5,6,7,8
D5	2,3	1,2,5,6,7,8
D6	2,3	1,2,5,6,7,8
D7	2,3	1,2,5,6,7,8

AGA (IgG): 5.9

Stool calprotectin: 14

Step3- Symptoms recurrence with GCP & symptoms improvement with GFD

First month

Month weeks	GI symptoms	Extra GI symptoms
W1 GCP	+++	+++++++
W2 GFD	++	++++++
W3 GFD	++	++++++
W4 GFD	++	++++++

Second month

Month weeks	GI symptoms	Extra GI symptoms
W1 GCP	+++	+++++++
W2 GFD	++	++++++
W3 GFD	++	++++++
W4 GFD	++	++++++

Third month

Month weeks	GI symptoms	Extra GI symptoms
W1 GCP	+++	+++++++
W2 GFD	++	++++++
W3 GFD	++	++++++
W4 GFD	++	++++++

Patients name: B

code: 6

Age: 28

Gender: *male* *female*

Address: Iran- Ahvaz

Accompany autoimmune diseases: (DM, Hashimoto thyroiditis, Vitiligo, Adison, Herpetiform dermatitis) *Yes ....* *No*

Family history of Celiac disease: *Yes* *No*

Family history of same symptoms in first degree: *Yes* *No*

GI Symptoms : *1. Epigastric pain/burning* *2. Post prandial fullness* *3. Early satiety*

Extra GI symptoms :

1.Fatigue & weakness *2.musculoskeletal pain* *3.headache* *4.allergic rhinitis* *5.deppression* *6.menstrual disorder*

7.disturbed sleep pattern *8.ataxia*

Serologic evaluation: *tTGA (IgA): 1* *Total IgA: 176* *AGA (IgG): 41.8* *Anti wheat specific (IgE): < 0.35 KUI/L*

Symptoms improvement

GI Symptoms : 1 2 3

Extra GI symptoms : 1 2 3 4 5 6 7 8

EGD report: *Mild antral erythema* *Marsh <1*

Step2- Symptoms recurrence with gluten reintroduction

First week

Week days	*GI symptoms*	*Extra GI symptoms*
D1	No	1
D2	No	1
D3	No	1
D4	No	1
D5	No	No
D6	No	No
D7	No	No

AGA (IgG): 2

Stool calprotectin: 10

Second week

Week days	*GI symptoms*	*Extra GI symptoms*
D1	No	No
D2	No	No
D3	No	No
D4	No	No
D5	No	No
D6	No	No
D7	No	No

Third week

Week days	*GI symptoms*	*Extra GI symptoms*
D1	No	No
D2	No	No
D3	No	No
D4	No	No
D5	No	No
D6	No	No
D7	No	No

AGA (IgG): 3

Stool calprotectin: 10

Step3- Symptoms recurrence with GCP & symptoms improvement with GFD

First month

Month weeks	*GI symptoms*	*Extra GI symptoms*
W1 GCP	+	++
W2 GFD	-	+
W3 GFD	-	-
W4 GFD	-	-

Second month

Month weeks	*GI symptoms*	*Extra GI symptoms*
W1 GCP	+	+++
W2 GFD	-	-
W3 GFD	-	-
W4 GFD	-	-

Third month

Month weeks	*GI symptoms*	*Extra GI symptoms*
W1 GCP	+	+++
W2 GFD	-	-
W3 GFD	-	-
W4 GFD	-	-

Patients name: B

code: 7

Age: 53

Gender: male female

Address: Iran- Qazvin

Accompany autoimmune diseases: (DM, Hashimoto thyroiditis, Vitiligo, Adison, Herpetiform dermatitis) *Yes ....* *No*

Family history of Celiac disease: *Yes* *No*

Family history of same symptoms in first degree: *Yes* *No*

GI Symptoms : *1. Epigastric pain/burning* *2. Post prandial fullness* *3. Early satiety*

Extra GI symptoms :

1.Fatigue & weakness *2.musculoskeletal pain* *3.headache* *4.allergic rhinitis* *5.deppression* *6.menstrual disorder* *7.disturbed sleep pattern* *8.ataxia*

Serologic evaluation: *tTGA (IgA): 4* *Total IgA: 162* *AGA (IgG): 29* *Anti wheat specific (IgE): < 0.35 KUI/L*

Symptoms improvement

GI Symptoms : 1 2 3

Extra GI symptoms : 1 2 3 4 5 6 7 8

EGD report: Mild antral erythema *Marsh 0*

Step2- Symptoms recurrence with gluten reintroduction

First week

Week days	GI symptoms	Extra GI symptoms
D1	No	1
D2	No	1
D3	No	1
D4	No	1
D5	No	1
D6	No	1
D7	No	1

AGA (IgG): 4.1

Stool calprotectin: 27.9

Second week

Week days	GI symptoms	Extra GI symptoms
D1	No	1
D2	No	1
D3	No	1
D4	No	1
D5	No	1
D6	No	1
D7	No	1

Third week

Week days	GI symptoms	Extra GI symptoms
D1	No	1
D2	No	1
D3	No	1
D4	No	1
D5	No	1
D6	No	1
D7	No	1

AGA (IgG):3.8

Stool calprotectin: 17.5

Step3- Symptoms recurrence with GCP & symptoms improvement with GFD

First month

Month weeks	GI symptoms	Extra GI symptoms
W1 GCP	-	+
W2 GFD	-	+
W3 GFD	-	+
W4 GFD	-	+

Second month

Month weeks	GI symptoms	Extra GI symptoms
W1 GCP	-	+
W2 GFD	-	+
W3 GFD	-	+
W4 GFD	-	+

Third month

Month weeks	GI symptoms	Extra GI symptoms
W1 GCP	-	+
W2 GFD	-	+
W3 GFD	-	+
W4 GFD	-	+

Patients name: B

code: 8

Age: 27

Gender: *male* *female*

Address: Iran- Tehran

Accompany autoimmune diseases: (DM, Hashimoto thyroiditis, Vitiligo, Adison, Herpetiform dermatitis) *Yes* .... *No*

Family history of Celiac disease: *Yes* *No*

Family history of same symptoms in first degree: *Yes* *No*

GI Symptoms : *1. Epigastric pain/burning* *2. Post prandial fullness* *3. Early satiety*

Extra GI symptoms :

1.Fatigue & weakness *2.musculoskeletal pain* *3.headache* *4.allergic rhinitis* *5.deppression* *6.menstrual disorder*

7.disturbed sleep pattern *8.ataxia*

Serologic evaluation: *tTGA (IgA): 6* *Total IgA: 165* *AGA (IgG): 6.5* *Anti wheat specific (IgE): < 0.35 KUI/L*

Symptoms improvement

GI Symptoms : 1 2 3

Extra GI symptoms : 1 2 3 4 5 6 7 8

EGD report: *Normal* *Marsh 0*

Step2- Symptoms recurrence with gluten reintroduction

First week

Week days	*GI symptoms*	*Extra GI symptoms*
D1	No	No
D2	No	No
D3	No	No
D4	No	No
D5	No	No
D6	No	No
D7	No	No

AGA (IgG): 5.5

Stool calprotectin: 10

Second week

Week days	*GI symptoms*	*Extra GI symptoms*
D1	No	No
D2	No	No
D3	No	No
D4	No	No
D5	No	No
D6	No	No
D7	No	No

Third week

Week days	*GI symptoms*	*Extra GI symptoms*
D1	No	No
D2	No	No
D3	No	No
D4	No	No
D5	No	No
D6	No	No
D7	No	No

AGA (IgG): 6.5

Stool calprotectin: 12

Step3- Symptoms recurrence with GCP & symptoms improvement with GFD

First month

Month weeks	*GI symptoms*	*Extra GI symptoms*
W1 GCP	+	-
W2 GFD	-	-
W3 GFD	-	-
W4 GFD	-	-

Second month

Month weeks	*GI symptoms*	*Extra GI symptoms*
W1 GCP	-	-
W2 GFD	-	-
W3 GFD	-	-
W4 GFD	-	-

Third month

Month weeks	*GI symptoms*	*Extra GI symptoms*
W1 GCP	-	-
W2 GFD	-	-
W3 GFD	-	-
W4 GFD	-	-

Patients name: B

code: 9

Age: 32

Gender: male female

Address: Iran- Tehran

Accompany autoimmune diseases: (DM, Hashimoto thyroiditis, Vitiligo, Adison, Herpetiform dermatitis) *Yes ....* *No*

Family history of Celiac disease: *Yes* *No*

Family history of same symptoms in first degree: *Yes* *No*

GI Symptoms : *1. Epigastric pain/burning* *2. Post prandial fullness* *3. Early satiety*

Extra GI symptoms :

1.Fatigue & weakness *2.musculoskeletal pain* *3.headache* *4.allergic rhinitis* *5.deppression* *6.menstrual disorder*

7.disturbed sleep pattern *8.ataxia*

Serologic evaluation: *tTGA (IgA): 5* *Total IgA: 185* *AGA (IgG): 4* *Anti wheat specific (IgE): < 0.35 KUI/L*

Symptoms improvement

GI Symptoms : 1 2 3

Extra GI symptoms : 1 2 3 4 5 6 7 8

EGD report: Normal *Marsh 0*

Step2- Symptoms recurrence with gluten reintroduction

First week

Week days	*GI symptoms*	*Extra GI symptoms*
D1	No	No
D2	No	No
D3	No	No
D4	No	No
D5	No	No
D6	No	No
D7	No	No

AGA (IgG): 5.5

Stool calprotectin: 12

Second week

Week days	*GI symptoms*	*Extra GI symptoms*
D1	No	No
D2	No	No
D3	No	No
D4	No	No
D5	No	No
D6	No	No
D7	No	No

Third week

Week days	*GI symptoms*	*Extra GI symptoms*
D1	No	No
D2	No	No
D3	No	No
D4	No	No
D5	No	No
D6	No	No
D7	No	No

AGA (IgG):5

Stool calprotectin: 9

Step3- Symptoms recurrence with GCP & symptoms improvement with GFD

First month

Month weeks	*GI symptoms*	*Extra GI symptoms*
W1 GCP	-	-
W2 GFD	+	-
W3 GFD	-	-
W4 GFD	+	-

Second month

Month weeks	*GI symptoms*	*Extra GI symptoms*
W1 GCP	-	-
W2 GFD	-	-
W3 GFD	-	-
W4 GFD	-	-

Third month

Month weeks	*GI symptoms*	*Extra GI symptoms*
W1 GCP	+	-
W2 GFD	+	-
W3 GFD	-	-
W4 GFD	-	-

Patients name: **B**

code: 10

Age: 35

Gender: *male* *female*

Address: Iran- Arak

Accompany autoimmune diseases: (DM, Hashimoto thyroiditis, Vitiligo, Adison, Herpetiform dermatitis) *Yes* .... *No*

Family history of Celiac disease: *Yes* *No*

Family history of same symptoms in first degree: *Yes* *No*

GI Symptoms : *1. Epigastric pain/burning* *2. Post prandial fullness* *3. Early satiety*

Extra GI symptoms :

1.Fatigue & weakness *2.musculoskeletal pain* *3.headache* *4.allergic rhinitis* *5.deppression* *6.menstrual disorder*

7.disturbed sleep pattern *8.ataxia*

Serologic evaluation: *tTGA (IgA): 5* *Total IgA: 200* *AGA (IgG): 8* *Anti wheat specific (IgE): < 0.35 KUI/L*

Symptoms improvement

GI Symptoms : 1 2 3

Extra GI symptoms : 1 2 3 4 5 6 7 8

EGD report: *Mild antral erythema* *Marsh 0*

Step2- Symptoms recurrence with gluten reintroduction

First week

Week days	*GI symptoms*	*Extra GI symptoms*
D1	No	No
D2	No	No
D3	No	No
D4	No	No
D5	No	No
D6	No	No
D7	No	No

AGA (IgG): 5.5

Stool calprotectin: 14

Second week

Week days	*GI symptoms*	*Extra GI symptoms*
D1	No	No
D2	No	No
D3	No	No
D4	No	No
D5	No	No
D6	No	No
D7	No	No

Third week

Week days	*GI symptoms*	*Extra GI symptoms*
D1	No	No
D2	No	No
D3	No	No
D4	No	No
D5	No	No
D6	No	No
D7	No	No

AGA (IgG): 5

Stool calprotectin: 12

Step3- Symptoms recurrence with GCP & symptoms improvement with GFD

First month

Month weeks	*GI symptoms*	*Extra GI symptoms*
W1 GCP	+	-
W2 GFD	+	-
W3 GFD	+	-
W4 GFD	-	-

Second month

Month weeks	*GI symptoms*	*Extra GI symptoms*
W1 GCP	++	-
W2 GFD	++	-
W3 GFD	-	-
W4 GFD	-	-

Third month

Month weeks	*GI symptoms*	*Extra GI symptoms*
W1 GCP	+	-
W2 GFD	+	-
W3 GFD	-	-
W4 GFD	-	-

Patients name: B

code: 11

Age: 27

Gender: *male* *female*

Address: Iran- Tehran

Accompany autoimmune diseases: (DM, Hashimoto thyroiditis, Vitiligo, Adison, Herpetiform dermatitis) *Yes ....* *No*

Family history of Celiac disease: *Yes* *No*

Family history of same symptoms in first degree: *Yes* *No*

GI Symptoms : *1. Epigastric pain/burning* *2. Post prandial fullness* *3. Early satiety*

Extra GI symptoms :

1.Fatigue & weakness *2.musculoskeletal pain* *3.headache* *4.allergic rhinitis* *5.deppression* *6.menstrual disorder*

7.disturbed sleep pattern *8.ataxia*

Serologic evaluation: *tTGA (IgA): 6* *Total IgA: 172* *AGA (IgG): 6* *Anti wheat specific (IgE): < 0.35 KUI/L*

Symptoms improvement

GI Symptoms : 1 2 3

Extra GI symptoms : 1 2 3 4 5 6 7 8

EGD report: Mild antral erythema *Marsh 0*

Step2- Symptoms recurrence with gluten reintroduction

First week		
Week days	*GI symptoms*	*Extra GI symptoms*
D1	No	No
D2	No	No
D3	No	No
D4	No	No
D5	No	No
D6	No	No
D7	No	No

AGA (IgG): 5.5

Stool calprotectin: 10

Second week		
Week days	*GI symptoms*	*Extra GI symptoms*
D1	No	No
D2	No	No
D3	No	No
D4	No	No
D5	No	No
D6	No	No
D7	No	No

Third week		
Week days	*GI symptoms*	*Extra GI symptoms*
D1	No	No
D2	No	No
D3	No	No
D4	No	No
D5	No	No
D6	No	No
D7	No	No

AGA (IgG): 6.5

Stool calprotectin: 12

Step3- Symptoms recurrence with GCP & symptoms improvement with GFD

First month		
Month weeks	*GI symptoms*	*Extra GI symptoms*
W1 GCP	+	-
W2 GFD	-	-
W3 GFD	+	-
W4 GFD	-	-

Second month		
Month weeks	*GI symptoms*	*Extra GI symptoms*
W1 GCP	-	-
W2 GFD	+	-
W3 GFD	-	-
W4 GFD	-	-

Third month		
Month weeks	*GI symptoms*	*Extra GI symptoms*
W1 GCP	-	-
W2 GFD	+	-
W3 GFD	+	-
W4 GFD	-	-

Patients name: B

code: 12

Age: 40

Gender: male *female*

Address: Iran- Qaemshahr

Accompany autoimmune diseases: (DM, Hashimoto thyroiditis, Vitiligo, Adison, Herpetiform dermatitis) *Yes ....* *No*

Family history of Celiac disease: *Yes* *No*

Family history of same symptoms in first degree: *Yes* *No*

GI Symptoms : *1. Epigastric pain/burning* *2. Post prandial fullness* *3. Early satiety*

Extra GI symptoms :

1.Fatigue & weakness *2.musculoskeletal pain* *3.headache* *4.allergic rhinitis* *5.deppression* *6.menstrual disorder*

7.disturbed sleep pattern *8.ataxia*

Serologic evaluation: *tTGA (IgA): 1* *Total IgA: 138* *AGA (IgG): 2* *Anti wheat specific (IgE): < 0.35 KUI/L*

Symptoms improvement

GI Symptoms : 1 2 3

Extra GI symptoms : 1 2 3 4 5 6 7 8

EGD report: Normal *Marsh <1*

Step2- Symptoms recurrence with gluten reintroduction

First week

Week days	*GI symptoms*	*Extra GI symptoms*
D1	*No*	*5*
D2	*No*	*5*
D3	*No*	*5*
D4	*No*	*5*
D5	*No*	*5*
D6	*No*	*5*
D7	*No*	*5*

AGA (IgG): 3

Stool calprotectin: 35

Second week

Week days	*GI symptoms*	*Extra GI symptoms*
D1	*No*	*5*
D2	*No*	*5*
D3	*No*	*5*
D4	*No*	*5*
D5	*No*	*5*
D6	*No*	*5*
D7	*No*	*5*

Third week

Week days	*GI symptoms*	*Extra GI symptoms*
D1	*No*	*5*
D2	*No*	*5*
D3	*No*	*5*
D4	*No*	*5*
D5	*No*	*5*
D6	*No*	*5*
D7	*No*	*5*

AGA (IgG): 3.8

Stool calprotectin: 10

Step3- Symptoms recurrence with GCP & symptoms improvement with GFD

First month

Month weeks	*GI symptoms*	*Extra GI symptoms*
W1 GCP	+	++
W2 GFD	-	+
W3 GFD	-	+
W4 GFD	-	+

Second month

Month weeks	*GI symptoms*	*Extra GI symptoms*
W1 GCP	++	++
W2 GFD	-	+
W3 GFD	-	+
W4 GFD	-	+

Third month

Month weeks	*GI symptoms*	*Extra GI symptoms*
W1 GCP	++	++
W2 GFD	-	+
W3 GFD	-	+
W4 GFD	-	+

Patients name: B

code: 13

Age: 50

Gender: *male* *female*

Address: Iran- Noshahr

Accompany autoimmune diseases: (DM, Hashimoto thyroiditis, Vitiligo, Adison, Herpetiform dermatitis) *Yes ....* *No*

Family history of Celiac disease: *Yes* *No*

Family history of same symptoms in first degree: *Yes* *No*

GI Symptoms : *1. Epigastric pain/burning* *2. Post prandial fullness* *3. Early satiety*

Extra GI symptoms :

1.Fatigue & weakness *2.musculoskeletal pain* *3.headache* *4.allergic rhinitis* *5.deppression* *6.menstrual disorder*

7.disturbed sleep pattern *8.ataxia*

Serologic evaluation: *tTGA (IgA): 6* *Total IgA: 174* *AGA (IgG): 6.5* *Anti wheat specific (IgE): < 0.35 KUI/L*

Symptoms improvement

GI Symptoms : 1 2 3

Extra GI symptoms : 1 2 3 4 5 6 7 8

EGD report: *Mild antral erythema* *Marsh 0*

Step2- Symptoms recurrence with gluten reintroduction

First week

Week days	*GI symptoms*	*Extra GI symptoms*
D1	No	No
D2	No	No
D3	No	No
D4	No	No
D5	No	No
D6	No	No
D7	No	No

AGA (IgG): 6

Stool calprotectin: 14

Second week

Week days	*GI symptoms*	*Extra GI symptoms*
D1	No	No
D2	No	No
D3	No	No
D4	No	No
D5	No	No
D6	No	No
D7	No	No

Third week

Week days	*GI symptoms*	*Extra GI symptoms*
D1	2	No
D2	2	No
D3	2	No
D4	2	No
D5	2	No
D6	No	No
D7	No	No

AGA (IgG):5.5

Stool calprotectin: 20

Step3- Symptoms recurrence with GCP & symptoms improvement with GFD

First month

Month weeks	*GI symptoms*	*Extra GI symptoms*
W1 GCP	++	-
W2 GFD	-	-
W3 GFD	-	-
W4 GFD	-	-

Second month

Month weeks	*GI symptoms*	*Extra GI symptoms*
W1 GCP	+	-
W2 GFD	+	-
W3 GFD	-	-
W4 GFD	-	-

Third month

Month weeks	*GI symptoms*	*Extra GI symptoms*
W1 GCP	+	-
W2 GFD	-	-
W3 GFD	-	-
W4 GFD	-	-

Patients name: B

code: 14

Age: 25

Gender: <u>male</u> female

Address: Iran- Karaj

Accompany autoimmune diseases: (DM, Hashimoto thyroiditis, Vitiligo, Adison, Herpetiform dermatitis) *Yes ....* *<u>No</u>*

Family history of Celiac disease: *Yes* *<u>No</u>*

Family history of same symptoms in first degree: *Yes* *<u>No</u>*

GI Symptoms : *<u>1. Epigastric pain/burning</u>* *<u>2. Post prandial fullness</u>* *3. Early satiety*

Extra GI symptoms :

1.Fatigue & weakness *2.musculoskeletal pain* *3.headache* *4.allergic rhinitis* *5.deppression* *6.menstrual disorder*

7.disturbed sleep pattern *8.ataxia*

Serologic evaluation: *tTGA (IgA): 3.3* *Total IgA: 185* *AGA (IgG): <u>21</u>* *Anti wheat specific (IgE): < 0.35 KUI/L*

Symptoms improvement

GI Symptoms : <u>1 2</u> 3

Extra GI symptoms : 1 2 3 4 5 6 7 8

EGD report: Normal *Marsh 0*

Step2- Symptoms recurrence with gluten reintroduction

First week

Week days	*GI symptoms*	*Extra GI symptoms*
**D1**	*No*	*No*
**D2**	*No*	*No*
**D3**	*No*	*No*
**D4**	*No*	*No*
**D5**	*No*	*No*
**D6**	*No*	*No*
**D7**	*No*	*No*

AGA (IgG): 5.4

Stool calprotectin: <u>54</u>

Second week

Week days	*GI symptoms*	*Extra GI symptoms*
**D1**	*No*	*No*
**D2**	*No*	*No*
**D3**	*No*	*No*
**D4**	*No*	*No*
**D5**	*No*	*No*
**D6**	*No*	*No*
**D7**	*No*	*No*

Third week

Week days	*GI symptoms*	*Extra GI symptoms*
**D1**	*2*	*No*
**D2**	*2*	*No*
**D3**	*No*	*No*
**D4**	*No*	*No*
**D5**	*No*	*No*
**D6**	*No*	*No*
**D7**	*No*	*No*

AGA (IgG):5.5

Stool calprotectin: <u>60</u>

Step3- Symptoms recurrence with GCP & symptoms improvement with GFD

First month

Month weeks	*GI symptoms*	*Extra GI symptoms*
**W1 GCP**	++	-
**W2 GFD**	+	-
**W3 GFD**	-	-
**W4 GFD**	-	-

Second month

Month weeks	*GI symptoms*	*Extra GI symptoms*
**W1 GCP**	-	-
**W2 GFD**	+	-
**W3 GFD**	+	-
**W4 GFD**	-	-

Third month

Month weeks	*GI symptoms*	*Extra GI symptoms*
**W1 GCP**	++	-
**W2 GFD**	+	-
**W3 GFD**	+	-
**W4 GFD**	-	-

Patients name: A

code: 15

Age: 35

Gender: male female

Address: Iran- Karaj

Accompany autoimmune diseases: (DM, Hashimoto thyroiditis, Vitiligo, Adison, Herpetiform dermatitis) *Yes ....* *No*

Family history of Celiac disease: *Yes* *No*

Family history of same symptoms in first degree: *Yes* *No*

GI Symptoms : *1. Epigastric pain/burning* *2. Post prandial fullness* *3. Early satiety*

Extra GI symptoms :

1.Fatigue & weakness *2.musculoskeletal pain* *3.headache* *4.allergic rhinitis* *5.deppression* *6.menstrual disorder*

7.disturbed sleep pattern *8.ataxia*

Serologic evaluation: *tTGA (IgA): 5.5* *Total IgA: 170* *AGA (IgG): 5* *Anti wheat specific (IgE): < 0.35 KUI/L*

Symptoms improvement

GI Symptoms : 1 2 3

Extra GI symptoms : 1 2 3 4 5 6 7 8

EGD report: Mild patchy antral erythema *Marsh 0*

Step2- Symptoms recurrence with gluten reintroduction

First week		
Week days	*GI symptoms*	*Extra GI symptoms*
D1	1,2	No
D2	1,2	No
D3	1,2	No
D4	1,2	No
D5	1,2	No
D6	1,2	No
D7	1,2	No

AGA (IgG): 4.5

Stool calprotectin: 14

Second week		
Week days	*GI symptoms*	*Extra GI symptoms*
D1	1,2	No
D2	1,2	No
D3	1,2	No
D4	1,2	No
D5	No	No
D6	No	No
D7	No	No

Third week		
Week days	*GI symptoms*	*Extra GI symptoms*
D1	1,2	No
D2	1,2	No
D3	1,2	No
D4	No	No
D5	1,2	No
D6	No	No
D7	No	No

AGA (IgG): 6

Stool calprotectin: 24

Step3- Symptoms recurrence with GCP & symptoms improvement with GFD

First month		
Month weeks	*GI symptoms*	*Extra GI symptoms*
W1 GCP	++	-
W2 GFD	-	-
W3 GFD	+	-
W4 GFD	+	-

Second month		
Month weeks	*GI symptoms*	*Extra GI symptoms*
W1 GCP	++	-
W2 GFD	+	-
W3 GFD	+	-
W4 GFD	+	-

Third month		
Month weeks	*GI symptoms*	*Extra GI symptoms*
W1 GCP	+	-
W2 GFD	+	-
W3 GFD	++	-
W4 GFD	+	-

Patients name: A

code: 16

Age: 25

Gender: male *female*

Address: Iran- Tehran

Accompany autoimmune diseases: (DM, Hashimoto thyroiditis, Vitiligo, Adison, Herpetiform dermatitis) Yes .... No

Family history of Celiac disease: Yes No

Family history of same symptoms in first degree: Yes No

GI Symptoms : *1. Epigastric pain/burning__* *2. Post prandial fullness* *3. Early satiety*

Extra GI symptoms :

1.Fatigue & weakness *2.musculoskeletal pain* *3.headache* *4.allergic rhinitis* *5.deppression* *6.menstrual disorder*

7.disturbed sleep pattern *8.ataxia*

Serologic evaluation: *tTGA (IgA): 4.5* *Total IgA: 170* *AGA (IgG): 4.8* *Anti wheat specific (IgE): < 0.35 KUI/L*

Symptoms improvement

GI Symptoms : 1 2 3

Extra GI symptoms : 1 2 3 4 5 6 7 8

EGD report: Normal *Marsh 0*

Step2- Symptoms recurrence with gluten reintroduction

First week

Week days	GI symptoms	Extra GI symptoms
D1	No	No
D2	No	No
D3	No	No
D4	No	No
D5	No	No
D6	No	No
D7	No	No

AGA (IgG): 4.5

Stool calprotectin: 20

Second week

Week days	GI symptoms	Extra GI symptoms
D1	No	No
D2	No	No
D3	No	No
D4	No	No
D5	No	No
D6	No	No
D7	No	No

Third week

Week days	GI symptoms	Extra GI symptoms
D1	No	No
D2	No	No
D3	No	No
D4	No	No
D5	No	No
D6	No	No
D7	No	No

AGA (IgG): 5

Stool calprotectin: 12

Step3- Symptoms recurrence with GCP & symptoms improvement with GFD

First month

Month weeks	GI symptoms	Extra GI symptoms
W1 GCP	++	-
W2 GFD	+	-
W3 GFD	+	-
W4 GFD	-	-

Second month

Month weeks	GI symptoms	Extra GI symptoms
W1 GCP	+	-
W2 GFD	+	-
W3 GFD	-	-
W4 GFD	-	-

Third month

Month weeks	GI symptoms	Extra GI symptoms
W1 GCP	+	-
W2 GFD	+	-
W3 GFD	+	-
W4 GFD	-	-

Patients name: A

code: 17

Age: 47

Gender: *male* female

Address: Iran- Qom

Accompany autoimmune diseases: (DM, Hashimoto thyroiditis, Vitiligo, Adison, Herpetiform dermatitis) Yes .... No

Family history of Celiac disease: Yes No

Family history of same symptoms in first degree: Yes No

GI Symptoms : *1. Epigastric pain/burning* *2. Post prandial fullness* *3. Early satiety*

Extra GI symptoms :

1.Fatigue & weakness *2.musculoskeletal pain* *3.headache* *4.allergic rhinitis* *5.deppression* *6.menstrual disorder*

7.disturbed sleep pattern *8.ataxia*

Serologic evaluation: *tTGA (IgA):* 5 *Total IgA:* 175 *AGA (IgG):* 8.5 *Anti wheat specific (IgE):* < 0.35 KUI/L

Symptoms improvement

GI Symptoms : 1 2 3

Extra GI symptoms : 1 2 3 4 5 6 7 8

EGD report: Normal *Marsh* 0

Step2- Symptoms recurrence with gluten reintroduction

First week		
Week days	*GI symptoms*	*Extra GI symptoms*
D1	No	No
D2	No	No
D3	No	No
D4	No	No
D5	No	No
D6	No	No
D7	No	No

AGA (IgG): 4

Stool calprotectin: 8

Second week		
Week days	*GI symptoms*	*Extra GI symptoms*
D1	No	No
D2	No	No
D3	No	No
D4	No	No
D5	No	No
D6	No	No
D7	No	No

Third week		
Week days	*GI symptoms*	*Extra GI symptoms*
D1	1,2	No
D2	1,2	No
D3	2	No
D4	2	No
D5	No	No
D6	No	No
D7	No	No

AGA (IgG): 3.8

Stool calprotectin: 12

Step3- Symptoms recurrence with GCP & symptoms improvement with GFD

First month		
Month weeks	*GI symptoms*	*Extra GI symptoms*
W1 GCP	++	-
W2 GFD	+	-
W3 GFD	+	-
W4 GFD	-	-

Second month		
Month weeks	*GI symptoms*	*Extra GI symptoms*
W1 GCP	+	-
W2 GFD	+	-
W3 GFD	-	-
W4 GFD	-	-

Third month		
Month weeks	*GI symptoms*	*Extra GI symptoms*
W1 GCP	+	-
W2 GFD	+	-
W3 GFD	-	-
W4 GFD	-	-

Patients name: A

code: 18

Age: 42

Gender: *male* *female*

Address: Iran- Tehran

Accompany autoimmune diseases: (DM, Hashimoto thyroiditis, Vitiligo, Adison, Herpetiform dermatitis) *Yes ....* *No*

Family history of Celiac disease: *Yes* *No*

Family history of same symptoms in first degree: *Yes* *No*

GI Symptoms : *1. Epigastric pain/burning* *2. Post prandial fullness* *3. Early satiety*

Extra GI symptoms :

1.Fatigue & weakness *2.musculoskeletal pain* *3.headache* *4.allergic rhinitis* *5.deppression* *6.menstrual disorder*

7.disturbed sleep pattern *8.ataxia*

Serologic evaluation: *tTGA (IgA): 6* *Total IgA: 180* *AGA (IgG): 8* *Anti wheat specific (IgE): < 0.35 KUI/L*

Symptoms improvement

GI Symptoms : 1 2 3

Extra GI symptoms : 1 2 3 4 5 6 7 8

EGD report: Normal *Marsh 0*

Step2- Symptoms recurrence with gluten reintroduction

First week

Week days	GI symptoms	Extra GI symptoms
D1	1	No
D2	1	No
D3	1	No
D4	No	No
D5	1	No
D6	1	No
D7	No	No

AGA (IgG): 5

Stool calprotectin: 8

Second week

Week days	GI symptoms	Extra GI symptoms
D1	No	No
D2	No	No
D3	No	No
D4	No	No
D5	No	No
D6	No	No
D7	No	No

Third week

Week days	GI symptoms	Extra GI symptoms
D1	1	No
D2	1	No
D3	No	No
D4	No	No
D5	1	No
D6	No	No
D7	No	No

AGA (IgG): 4.2

Stool calprotectin: 10

Step3- Symptoms recurrence with GCP & symptoms improvement with GFD

First month

Month weeks	GI symptoms	Extra GI symptoms
W1 GCP	+	-
W2 GFD	-	-
W3 GFD	+	-
W4 GFD	-	-

Second month

Month weeks	GI symptoms	Extra GI symptoms
W1 GCP	+	-
W2 GFD	+	-
W3 GFD	-	-
W4 GFD	-	-

Third month

Month weeks	GI symptoms	Extra GI symptoms
W1 GCP	-	-
W2 GFD	+	-
W3 GFD	-	-
W4 GFD	+	-

Patients name: A **code:** 19

Age: 33

Gender: *male* *female*

Address: Iran- Tehran

Accompany autoimmune diseases: (DM, Hashimoto thyroiditis, Vitiligo, Adison, Herpetiform dermatitis) *Yes ....* *No*

Family history of Celiac disease: *Yes* *No*

Family history of same symptoms in first degree: *Yes* *No*

GI Symptoms : *1. Epigastric pain/burning* *2. Post prandial fullness* *3. Early satiety*

Extra GI symptoms :

1.Fatigue & weakness *2.musculoskeletal pain* *3.headache* *4.allergic rhinitis* *5.deppression* *6.menstrual disorder*

7.disturbed sleep pattern *8.ataxia*

Serologic evaluation: *tTGA (IgA): 8* *Total IgA: 162* *AGA (IgG): 5.5* *Anti wheat specific (IgE): < 0.35 KUI/L*

Symptoms improvement

GI Symptoms : 1 2 3

Extra GI symptoms : 1 2 3 4 5 6 7 8

EGD report: *Normal Mild hiatal hernia* *Marsh 0*

Step2- Symptoms recurrence with gluten reintroduction

First week

Week days	GI symptoms	Extra GI symptoms
D1	1	No
D2	1	No
D3	1	No
D4	No	No
D5	No	No
D6	No	No
D7	No	No

AGA (IgG): 8.5

Stool calprotectin: 8

Second week

Week days	GI symptoms	Extra GI symptoms
D1	No	No
D2	No	No
D3	No	No
D4	No	No
D5	1	No
D6	No	No
D7	No	No

Third week

Week days	GI symptoms	Extra GI symptoms
D1	No	No
D2	1	No
D3	No	No
D4	No	No
D5	1	No
D6	No	No
D7	No	No

AGA (IgG): 5

Stool calprotectin: 10

Step3- Symptoms recurrence with GCP & symptoms improvement with GFD

First month

Month weeks	GI symptoms	Extra GI symptoms
W1 GCP	-	-
W2 GFD	-	-
W3 GFD	+	-
W4 GFD	-	-

Second month

Month weeks	GI symptoms	Extra GI symptoms
W1 GCP	+	-
W2 GFD	+	-
W3 GFD	-	-
W4 GFD	-	-

Third month

Month weeks	GI symptoms	Extra GI symptoms
W1 GCP	-	-
W2 GFD	+	-
W3 GFD	-	-
W4 GFD	+	-

Patients name: A

code: 20

Age: 30

Gender: male female

Address: Iran- Tehran

Accompany autoimmune diseases: (DM, Hashimoto thyroiditis, Vitiligo, Adison, Herpetiform dermatitis) *Yes ....* *No*

Family history of Celiac disease: *Yes* *No*

Family history of same symptoms in first degree: *Yes* *No*

GI Symptoms : *1. Epigastric pain/burning* *2. Post prandial fullness* *3. Early satiety*

Extra GI symptoms :

1.Fatigue & weakness *2.musculoskeletal pain* *3.headache* *4.allergic rhinitis* *5.deppression* *6.menstrual disorder*

7.disturbed sleep pattern *8.ataxia*

Serologic evaluation: *tTGA (IgA): 3.3* *Total IgA: 184* *AGA (IgG): 8* *Anti wheat specific (IgE): < 0.35 KUI/L*

Symptoms improvement

GI Symptoms : 1 2 3

Extra GI symptoms : 1 2 3 4 5 6 7 8

EGD report: Normal *Marsh 0*

Step2- Symptoms recurrence with gluten reintroduction

First week

Week days	GI symptoms	Extra GI symptoms
D1	1..	No
D2	1	No
D3	1	No
D4	1	No
D5	No	No
D6	No	No
D7	No	No

AGA (IgG): 5.5

Stool calprotectin: 21

Second week

Week days	GI symptoms	Extra GI symptoms
D1	No	No
D2	1	No
D3	No	No
D4	No	No
D5	No	No
D6	No	No
D7	No	No

Third week

Week days	GI symptoms	Extra GI symptoms
D1	1	No
D2	1	No
D3	No	No
D4	1	No
D5	1	No
D6	1	No
D7	No	No

AGA (IgG): 6

Stool calprotectin: 24

Step3- Symptoms recurrence with GCP & symptoms improvement with GFD

First month

Month weeks	GI symptoms	Extra GI symptoms
W1 GCP	+	-
W2 GFD	-	-
W3 GFD	+	-
W4 GFD	-	-

Second month

Month weeks	GI symptoms	Extra GI symptoms
W1 GCP	+	-
W2 GFD	+	-
W3 GFD	-	-
W4 GFD	-	-

Third month

Month weeks	GI symptoms	Extra GI symptoms
W1 GCP	+	-
W2 GFD	+	-
W3 GFD	-	-
W4 GFD	+	-

Patients name: B

code: 21

Age: 29

Gender: *male* *female*

Address: Iran- Tehran

Accompany autoimmune diseases: (DM, Hashimoto thyroiditis, Vitiligo, Adison, Herpetiform dermatitis) *Yes ....* *No*

Family history of Celiac disease: *Yes* *No*

Family history of same symptoms in first degree: *Yes* *No*

GI Symptoms : *1. Epigastric pain/burning* *2. Post prandial fullness* *3. Early satiety*

Extra GI symptoms :

1.Fatigue & weakness *2.musculoskeletal pain* *3.headache* *4.allergic rhinitis* *5.deppression* *6.menstrual disorder*

7.disturbed sleep pattern *8.ataxia*

Serologic evaluation: *tTGA (IgA): 3.6* *Total IgA: 202* *AGA (IgG): 5.6* *Anti wheat specific (IgE): < 0.35 KUI/L*

Symptoms improvement

GI Symptoms : 1 2 3

Extra GI symptoms : 1 2 3 4 5 6 7 8

EGD report: Mild antral erythema+mild hiatal hernia *Marsh 0*

Step2- Symptoms recurrence with gluten reintroduction

First week

Week days	*GI symptoms*	*Extra GI symptoms*
D1	1	3,4
D2	1	3,4
D3	1	3,4
D4	1	3,4
D5	1	3,4
D6	1	3,4
D7	1	3,4

AGA (IgG): 2.3

Stool calprotectin: 8.3

Second week

Week days	*GI symptoms*	*Extra GI symptoms*
D1	No	3,4
D2	No	3,4
D3	No	3,4
D4	No	3,4
D5	No	3,4
D6	No	3,4
D7	No	3,4

Third week

Week days	*GI symptoms*	*Extra GI symptoms*
D1	No	1,3,4
D2	No	1,3,4
D3	No	1,3,4
D4	No	1,3,4
D5	No	1,3,4
D6	No	1,3,4
D7	No	1,3,4

AGA (IgG): 3.1

Stool calprotectin: 7.5

Step3- Symptoms recurrence with GCP & symptoms improvement with GFD

First month

Month weeks	*GI symptoms*	*Extra GI symptoms*
W1 GCP	+	+++
W2 GFD	-	++
W3 GFD	+	++
W4 GFD	-	++

Second month

Month weeks	*GI symptoms*	*Extra GI symptoms*
W1 GCP	+	+++
W2 GFD	+	+++
W3 GFD	-	+++
W4 GFD	-	+++

Third month

Month weeks	*GI symptoms*	*Extra GI symptoms*
W1 GCP	+	+++
W2 GFD	+	+++
W3 GFD	-	+++
W4 GFD	-	+++

Patients name: A

code: 22

Age: 35

Gender: *male* *female*

Address: Iran- Tehran

Accompany autoimmune diseases: (DM, Hashimoto thyroiditis, Vitiligo, Adison, Herpetiform dermatitis) *Yes ....* *No*

Family history of Celiac disease: *Yes* *No*

Family history of same symptoms in first degree: *Yes* *No*

GI Symptoms : *1. Epigastric pain/burning* *2. Post prandial fullness* *3. Early satiety*

Extra GI symptoms :

1.Fatigue & weakness *2.musculoskeletal pain* *3.headache* *4.allergic rhinitis* *5.deppression* *6.menstrual disorder*

7.disturbed sleep pattern *8.ataxia*

Serologic evaluation: *tTGA (IgA): 3.5* *Total IgA: 186* *AGA (IgG): 8.5* *Anti wheat specific (IgE): < 0.35 KUI/L*

Symptoms improvement

GI Symptoms : 1 2 3

Extra GI symptoms : 1 2 3 4 5 6 7 8

EGD report: *Mild patchy antral erythema* *Marsh 0*

Step2- Symptoms recurrence with gluten reintroduction

First week

Week days	*GI symptoms*	*Extra GI symptoms*
D1	No	1,2,3
D2	1	1,2,3
D3	No	1,2,3
D4	No	1,2,3
D5	No	1,2,3
D6	1	1,2,3
D7	No	1,2,3

AGA (IgG): 8

Stool calprotectin: 14

Second week

Week days	*GI symptoms*	*Extra GI symptoms*
D1	No	1,2,3
D2	No	1,2,3
D3	No	1,2,3
D4	No	1,2,3
D5	No	1,2,3
D6	No	1,2,3
D7	No	1,2,3

Third week

Week days	*GI symptoms*	*Extra GI symptoms*
D1	No	1,2,3
D2	No	1,2,3
D3	1	1,2,3
D4	No	1,2,3
D5	No	1,2,3
D6	No	1,2,3
D7	No	1,2,3

AGA (IgG): 7

Stool calprotectin: 12

Step3- Symptoms recurrence with GCP & symptoms improvement with GFD

First month

Month weeks	*GI symptoms*	*Extra GI symptoms*
W1 GCP	+	+++
W2 GFD	+	+++
W3 GFD	-	+++
W4 GFD	-	+++

Second month

Month weeks	*GI symptoms*	*Extra GI symptoms*
W1 GCP	-	+++
W2 GFD	-	+++
W3 GFD	+	+++
W4 GFD	-	+++

Third month

Month weeks	*GI symptoms*	*Extra GI symptoms*
W1 GCP	+	+++
W2 GFD	+	+++
W3 GFD	-	+++
W4 GFD	-	+++

Patients name: A

code: 23

Age: 32

Gender: male female

Address: Iran- Tehran

Accompany autoimmune diseases: (DM, Hashimoto thyroiditis, Vitiligo, Adison, Herpetiform dermatitis) *Yes ....* *No*

Family history of Celiac disease: *Yes* *No*

Family history of same symptoms in first degree: *Yes* *No*

GI Symptoms : *1. Epigastric pain/burning* *2. Post prandial fullness* *3. Early satiety*

Extra GI symptoms :

1.Fatigue & weakness *2.musculoskeletal pain* *3.headache* *4.allergic rhinitis* *5.deppression* *6.menstrual disorder*

7.disturbed sleep pattern *8.ataxia*

Serologic evaluation: *tTGA (IgA): 5.5* *Total IgA: 162* *AGA (IgG): 6* *Anti wheat specific (IgE): < 0.35 KUI/L*

Symptoms improvement

GI Symptoms : 1 2 3

Extra GI symptoms : 1 2 3 4 5 6 7 8

EGD report: Normal *Marsh 0*

Step2- Symptoms recurrence with gluten reintroduction

First week		
Week days	*GI symptoms*	*Extra GI symptoms*
D1	No	No
D2	No	No
D3	No	No
D4	No	No
D5	No	No
D6	No	No
D7	No	No

AGA (IgG): 5.4

Stool calprotectin: 10

Second week		
Week days	*GI symptoms*	*Extra GI symptoms*
D1	No	No
D2	No	No
D3	No	No
D4	No	No
D5	No	No
D6	No	No
D7	No	No

Third week		
Week days	*GI symptoms*	*Extra GI symptoms*
D1	1	No
D2	1	No
D3	1	No
D4	1	No
D5	No	No
D6	No	No
D7	No	No

AGA (IgG):5.2

Stool calprotectin: 12

Step3- Symptoms recurrence with GCP & symptoms improvement with GFD

First month		
Month weeks	*GI symptoms*	*Extra GI symptoms*
W1 GCP	-	-
W2 GFD	+	-
W3 GFD	-	-
W4 GFD	+	-

Second month		
Month weeks	*GI symptoms*	*Extra GI symptoms*
W1 GCP	+	-
W2 GFD	+	-
W3 GFD	-	-
W4 GFD	-	-

Third month		
Month weeks	*GI symptoms*	*Extra GI symptoms*
W1 GCP	+	-
W2 GFD	+	-
W3 GFD	-	-
W4 GFD	-	-

Patients name: A

code: 24

Age: 35

Gender: male female

Address: Iran- Qazvin

Accompany autoimmune diseases: (DM, Hashimoto thyroiditis, Vitiligo, Adison, Herpetiform dermatitis) *Yes ....* *No*

Family history of Celiac disease: *Yes* *No*

Family history of same symptoms in first degree: *Yes* *No*

GI Symptoms : *1. Epigastric pain/burning* *2. Post prandial fullness* *3. Early satiety*

Extra GI symptoms :

1.Fatigue & weakness *2.musculoskeletal pain* *3.headache* *4.allergic rhinitis* *5.deppression* *6.menstrual disorder*

7.disturbed sleep pattern *8.ataxia*

Serologic evaluation: *tTGA (IgA): 4* *Total IgA: 165* *AGA (IgG): 6* *Anti wheat specific (IgE): < 0.35 KUI/L*

Symptoms improvement

GI Symptoms : 1 2 3

Extra GI symptoms : 1 2 3 4 5 6 7 8

EGD report: *Mild patchy antral erythema* *Marsh 0*

Step2- Symptoms recurrence with gluten reintroduction

First week

Week days	*GI symptoms*	*Extra GI symptoms*
D1	No	No
D2	No	No
D3	No	No
D4	No	No
D5	1	No
D6	No	No
D7	No	No

AGA (IgG): 5.5

Stool calprotectin: 12

Second week

Week days	*GI symptoms*	*Extra GI symptoms*
D1	No	No
D2	No	No
D3	No	No
D4	No	No
D5	No	No
D6	No	No
D7	No	No

Third week

Week days	*GI symptoms*	*Extra GI symptoms*
D1	No	No
D2	1	No
D3	No	No
D4	No	No
D5	No	No
D6	No	No
D7	1	No

AGA (IgG):6

Stool calprotectin: 14

Step3- Symptoms recurrence with GCP & symptoms improvement with GFD

First month

Month weeks	*GI symptoms*	*Extra GI symptoms*
W1 GCP	+	-
W2 GFD	-	-
W3 GFD	-	-
W4 GFD	-	-

Second month

Month weeks	*GI symptoms*	*Extra GI symptoms*
W1 GCP	+	-
W2 GFD	+	-
W3 GFD	+	-
W4 GFD	-	-

Third month

Month weeks	*GI symptoms*	*Extra GI symptoms*
W1 GCP	+	-
W2 GFD	+	-
W3 GFD	-	-
W4 GFD	-	-

Patients name: A code: 25

Age: 52

Gender: male *female*

Address: Iran- Tehran

Accompany autoimmune diseases: (DM, Hashimoto thyroiditis, Vitiligo, Adison, Herpetiform dermatitis) Yes .... No

Family history of Celiac disease: Yes No

Family history of same symptoms in first degree: Yes No

GI Symptoms : *1. Epigastric pain/burning* *2. Post prandial fullness* *3. Early satiety*

Extra GI symptoms :

1.Fatigue & weakness *2.musculoskeletal pain* *3.headache* *4.allergic rhinitis* *5.deppression* *6.menstrual disorder*

7.disturbed sleep pattern *8.ataxia*

Serologic evaluation: *tTGA (IgA):* 3.5 *Total IgA:* 174 *AGA (IgG):* 4.3 *Anti wheat specific (IgE):* < 0.35 KUI/L

Symptoms improvement

GI Symptoms : 1 2 3

Extra GI symptoms : 1 2 3 4 5 6 7 8

EGD report: *Patchy antral erythema* *Marsh 0*

Step2- Symptoms recurrence with gluten reintroduction

First week		
Week days	*GI symptoms*	*Extra GI symptoms*
D1	1	No
D2	No	No
D3	1	No
D4	1	No
D5	1	No
D6	1	No
D7	No	No

AGA (IgG): 4.2

Stool calprotectin: 8.5

Second week		
Week days	*GI symptoms*	*Extra GI symptoms*
D1	No	No
D2	1	No
D3	No	No
D4	No	No
D5	No	No
D6	No	No
D7	No	No

Third week		
Week days	*GI symptoms*	*Extra GI symptoms*
D1	1	No
D2	1	No
D3	1	No
D4	1	No
D5	No	No
D6	No	No
D7	No	No

AGA (IgG): 6

Stool calprotectin: 6.5

Step3- Symptoms recurrence with GCP & symptoms improvement with GFD

First month		
Month weeks	*GI symptoms*	*Extra GI symptoms*
W1 GCP	+	-
W2 GFD	-	-
W3 GFD	+	-
W4 GFD	-	-

Second month		
Month weeks	*GI symptoms*	*Extra GI symptoms*
W1 GCP	-	-
W2 GFD	+	-
W3 GFD	-	-
W4 GFD	-	-

Third month		
Month weeks	*GI symptoms*	*Extra GI symptoms*
W1 GCP	-	-
W2 GFD	+	-
W3 GFD	+	-
W4 GFD	-	-

Patients name: B

code: 26

Age: 35

Gender: *male* *female*

Address: Iran- Tehran

Accompany autoimmune diseases: (DM, Hashimoto thyroiditis, Vitiligo, Adison, Herpetiform dermatitis) Yes .... No

Family history of Celiac disease: Yes No

Family history of same symptoms in first degree: Yes No

GI Symptoms : *1. Epigastric pain/burning* *2. Post prandial fullness* *3. Early satiety*

Extra GI symptoms :

1.Fatigue & weakness *2.musculoskeletal pain* *3.headache* *4.allergic rhinitis* *5.deppression* *6.menstrual disorder*

7.disturbed sleep pattern *8.ataxia*

Serologic evaluation: *tTGA (IgA): 3.5* *Total IgA: 185* *AGA (IgG): 4* *Anti wheat specific (IgE): < 0.35 KUI/L*

Symptoms improvement

GI Symptoms : 1 2 3

Extra GI symptoms : 1 2 3 4 5 6 7 8

EGD report: Normal *Marsh 0*

Step2- Symptoms recurrence with gluten reintroduction

First week

Week days	*GI symptoms*	*Extra GI symptoms*
D1	No	No
D2	No	No
D3	No	No
D4	No	No
D5	No	No
D6	No	No
D7	No	No

AGA (IgG): 4.8

Stool calprotectin: 6

Second week

Week days	*GI symptoms*	*Extra GI symptoms*
D1	No	No
D2	1	No
D3	No	No
D4	No	No
D5	No	No
D6	No	No
D7	No	No

Third week

Week days	*GI symptoms*	*Extra GI symptoms*
D1	No	No
D2	No	No
D3	No	No
D4	No	No
D5	No	No
D6	No	No
D7	No	No

AGA (IgG): 6.8

Stool calprotectin: 6.5

Step3- Symptoms recurrence with GCP & symptoms improvement with GFD

First month

Month weeks	*GI symptoms*	*Extra GI symptoms*
W1 GCP	-	-
W2 GFD	-	-
W3 GFD	+	-
W4 GFD	-	-

Second month

Month weeks	*GI symptoms*	*Extra GI symptoms*
W1 GCP	-	-
W2 GFD	+	-
W3 GFD	-	-
W4 GFD	-	-

Third month

Month weeks	*GI symptoms*	*Extra GI symptoms*
W1 GCP	+	-
W2 GFD	+	-
W3 GFD	-	-
W4 GFD	-	-

Patients name: A

code: 27

Age: 37

Gender: *male* *female*

Address: Iran- Tehran

Accompany autoimmune diseases: (DM, Hashimoto thyroiditis, Vitiligo, Adison, Herpetiform dermatitis) *Yes ....* *No*

Family history of Celiac disease: *Yes* *No*

Family history of same symptoms in first degree: *Yes* *No*

GI Symptoms : *1. Epigastric pain/burning* *2. Post prandial fullness* *3. Early satiety*

Extra GI symptoms :

1.Fatigue & weakness *2.musculoskeletal pain* *3.headache* *4.allergic rhinitis* *5.deppression* *6.menstrual disorder*

7.disturbed sleep pattern *8.ataxia*

Serologic evaluation: *tTGA (IgA): 4* *Total IgA: 170* *AGA (IgG): 5.5* *Anti wheat specific (IgE): < 0.35 KUI/L*

Symptoms improvement

GI Symptoms : 1 2 3

Extra GI symptoms : 1 2 3 4 5 6 7 8

EGD report: Normal *Marsh 0*

Step2- Symptoms recurrence with gluten reintroduction

First week

Week days	GI symptoms	Extra GI symptoms
D1	No	No
D2	No	No
D3	2	No
D4	2	No
D5	No	No
D6	No	No
D7	No	No

AGA (IgG): 5

Stool calprotectin: 10

Second week

Week days	GI symptoms	Extra GI symptoms
D1	No	No
D2	2	No
D3	No	No
D4	2	No
D5	2	No
D6	No	No
D7	No	No

Third week

Week days	GI symptoms	Extra GI symptoms
D1	2	No
D2	2	No
D3	2	No
D4	No	No
D5	No	No
D6	No	No
D7	No	No

AGA (IgG): 6.2

Stool calprotectin: 8

Step3- Symptoms recurrence with GCP & symptoms improvement with GFD

First month

Month weeks	GI symptoms	Extra GI symptoms
W1 GCP	++	-
W2 GFD	+	-
W3 GFD	+	-
W4 GFD	-	-

Second month

Month weeks	GI symptoms	Extra GI symptoms
W1 GCP	+	-
W2 GFD	++	-
W3 GFD	-	-
W4 GFD	-	-

Third month

Month weeks	GI symptoms	Extra GI symptoms
W1 GCP	+	-
W2 GFD	++	-
W3 GFD	+	-
W4 GFD	-	-

Normal labratory data values :

Total IgA (ELISA): 70-400 mg/dl

AGA (IgG) (ELISA): < 12 IU/ml = Negative

AGA (IgA) (ELISA): < 12 IU/ml = Negative

Anti tTG (IgG) (ELISA): < 10 IU/ml = Negative

Anti-Wheat IgE (International Rast Class) : <0.35KUA/L = Negative

Data analysis of two groups after step 2 (DBPC) for 27 patients

Table 1- Group A in all stages

Stage	Symptom Group A	1 NCGS	2 NCGS	3 NCGS	15	16	17	18	19	20	22	23	24	25	27
Before	GI	1	1	1	1	1	1	1	1	1	1	1	1	1	1
	weakness	1	1	1	0	0	0	0	0	0	1	0	0	0	0
	MS pain	1	1	0	0	0	0	0	0	0	1	0	0	0	0
	headache	1	1	1	0	0	0	0	0	0	1	0	0	0	0
1st week	GI	1	1	1	1	0.4	0.4	0.71	0.42	0.57	0.28	0	0.14	0.71	0.14
	weakness	1	1	1	0	0	0	0	0	0	1	0	0	0	0
	MS pain	1	1	0	0	0	0	0	0	0	1	0	0	0	0
	headache	1	1	1	0	0	0	0	0	0	1	0	0	0	0
2nd week	GI	0.28	0	0.4	0.77	0.4	0.4	0.4	0.14	0.14	0	0	0	0.14	0.21
	weakness	0.42	0.4	0.4	0	0	0	0	0	0	1	0	0	0	0
	MS pain	0.42	0.4	0	0	0	0	0	0	0	1	0	0	0	0
	headache	0.42	1	1	0	0	0	0	0	0	1	0	0	0	0
3rd week	GI	0.22	0	0.4	0.77	0.4	0.42	0.42	0.28	0.71	0.14	0.57	0.28	0.57	0.21
	weakness	0	0.4	0.4	0	0	0	0	0	0	1	0	0	0	0
	MS pain	0	0.4	0	0	0	0	0	0	0	1	0	0	0	0
	headache	0.42	1	1	0	0	0	0	0	0	1	0	0	0	0

Table 2- Group B in all stages

Stage	Symptom Group B	4 NCGS	5 NCGS	6	7	8	9	10	11	12	13	14	21	26
Before	GI	1	1	1	1	1	1	1	1	1	1	1	1	1
	weakness	0	1	1	1	0	0	0	0	1	0	0	1	0
	MS pain	0	1	1	0	0	0	0	0	1	0	0	0	0
	headache	0	1	0	0	0	0	0	0	0	0	0	1	0
1st week	GI	0.4	0.65	0.4	1	0.4	0.4	0.4	0.4	0.4	0.4	0.4	1	0.4
	weakness	0	1	0.57	0.4	0	0	0	0	0.4	0	0	0.4	0
	MS pain	0	1	0	0	0	0	0	0	0.4	0	0	0	0
	headache	0	0.4	0	0	0	0	0	0	0	0	0	1	0
2nd week	GI	0.4	0.65	0.4	1	0.4	0.4	0.4	0.4	0.4	0.4	0.4	0.4	0.14
	weakness	0	1	0	0.4	0	0	0	0	0.4	0	0	0.4	0
	MS pain	0	1	0	0	0	0	0	0	0.4	0	0	0	0
	headache	0	0.4	0	0	0	0	0	0	0	0	0	1	0
3rd week	GI	1	1	0.4	1	0.4	0.4	0.4	0.4	0.4	0.4	0.28	0.4	0.4
	weakness	0	1	0	0.4	0	0	0	0	0.4	0	0	1	0
	MS pain	0	1	0	0	0	0	0	0	0.4	0	0	0	0
	headache	0	0.4	0	0	0	0	0	0	0	0	0	1	0

Table 3, 4 - Comparison of Group A vs. Group B before and after treatment (before and after GFD)

Stage	Symptom Group A	1 NCGS	2 NCGS	3 NCGS	15	16	17	18	19	20	22	23	24	25	27
Before GFD	GI	1	1	1	1	0.7	0.7	0.85	0.71	0.78	0.64	0.5	0.57	0.85	0.57
	weakness	1	1	1	0	0	0	0	0	0	1	0	0	0	0
	MS pain	1	1	0	0	0	0	0	0	0	1	0	0	0	0
	headache	1	1	1	0	0	0	0	0	0	1	0	0	0	0
After GFD	GI	0.25	0	0.4	0.77	0.4	0.41	0.41	0.21	0.42	0.07	0.28	0.14	0.35	0.21
	weakness	0.21	0.4	0.4	0	0	0	0	0	0	1	0	0	0	0
	MS pain	0.21	0.4	0	0	0	0	0	0	0	1	0	0	0	0
	headache	0.42	1	1	0	0	0	0	0	0	1	0	0	0	0

Stage	Symptom Group B	4 NCGS	5 NCGS	6	7	8	9	10	11	12	13	14	21	26
Before GFD	GI	1	1	0.7	1	0.7	0.7	0.7	0.7	0.7	0.7	0.64	0.7	0.7
	weakness	0	1	0.5	0.7	0	0	0	0	0.7	0	0	1	0
	MS pain	0	1	0.5	0	0	0	0	0	0.7	0	0	0	0
	headache	0	0.7	0	0	0	0	0	0	0	0	0	1	0
After GFD	GI	0.4	0.65	0.4	1	0.4	0.4	0.4	0.4	0.4	0.4	0.4	0.7	0.27
	weakness	0	1	0.28	0.4	0	0	0	0	0.4	0	0	0.4	0
	MS pain	0	1	0	0	0	0	0	0	0.4	0	0	0	0
	headache	0	0.4	0	0	0	0	0	0	0	0	0	1	0

Table 5, 6 - Comparison of Group “NCGS” vs. “No NCGS” before and after treatment (before and after GFD)

Stage	Symptom NO NCGS	6	7	8	9	10	11	12	13	14	21	26	15	16	17	18	19	20	22	23	24	25	27
Before GFD	GI	0.7	1	0.7	0.7	0.7	0.7	0.7	0.7	0.64	0.7	0.7	1	0.7	0.7	0.85	0.71	0.78	0.64	0.5	0.57	0.85	0.57
	weakness	0.5	0.7	0	0	0	0	0.7	0	0	1	0	0	0	0	0	0	0	1	0	0	0	0
	MS pain	0.5	0	0	0	0	0	0.7	0	0	0	0	0	0	0	0	0	0	1	0	0	0	0
	headache	0	0	0	0	0	0	0	0	0	1	0	0	0	0	0	0	0	1	0	0	0	0
After GFD	GI	0.4	1	0.4	0.4	0.4	0.4	0.4	0.4	0.4	0.7	0.27	0.77	0.4	0.41	0.41	0.21	0.42	0.07	0.28	0.14	0.35	0.21
	weakness	0.28	0.4	0	0	0	0	0.4	0	0	0.4	0	0	0	0	0	0	0	1	0	0	0	0
	MS pain	0	0	0	0	0	0	0.4	0	0	0	0	0	0	0	0	0	0	1	0	0	0	0
	headache	0	0	0	0	0	0	0	0	0	1	0	0	0	0	0	0	0	1	0	0	0	0

Stage	Symptom NCGS	1	2	3	4	5
Before GFD	GI	1	1	1	1	1
	weakness	1	1	1	0	1
	MS pain	1	1	0	0	1
	headache	1	1	1	0	0.7
After GFD	GI	0.25	0	0.4	0.4	0.65
	weakness	0.21	0.4	0.4	0	1
	MS pain	0.21	0.4	0	0	1
	headache	0.42	1	1	0	0.4
